# Genetic basis of allochronic differentiation in the fall armyworm

**DOI:** 10.1186/s12862-017-0911-5

**Published:** 2017-03-06

**Authors:** Sabine Hänniger, Pascaline Dumas, Gerhard Schöfl, Steffi Gebauer-Jung, Heiko Vogel, Melanie Unbehend, David G. Heckel, Astrid T. Groot

**Affiliations:** 1 Max Planck Institute for Chemical Ecology, Entomology, Hans-Knöll-Str. 8, 07745 Jena, Germany; 2 Institute for Biodiversity and Ecosystem Dynamics, University of Amsterdam, Science Park 904, 1098 XH Amsterdam, The Netherlands; 3 DKMS Life Science Lab, Fiedlerstr, 34, 01307 Dresden, Germany

## Abstract

**Background:**

Very little is known on how changes in circadian rhythms evolve. The noctuid moth *Spodoptera frugiperda* (Lepidoptera: Noctuidae) consists of two strains that exhibit allochronic differentiation in their mating time, which acts as a premating isolation barrier between the strains. We investigated the genetic basis of the strain-specific timing differences to identify the molecular mechanisms of differentiation in circadian rhythms.

**Results:**

Through QTL analyses we identified one major Quantitative trait chromosome (QTC) underlying differentiation in circadian timing of mating activity. Using RADtags, we identified this QTC to be homologous to *Bombyx mori* C27, on which the clock gene *vrille* is located, which thus became the major candidate gene. In *S. frugiperda*, *vrille* showed strain-specific polymorphisms. Also, *vrille* expression differed significantly between the strains, with the rice-strain showing higher expression levels than the corn-strain. In addition, RT-qPCR experiments with the other main clock genes showed that *pdp1*, antagonist of *vrille* in the modulatory feedback loop of the circadian clock, showed higher expression levels in the rice-strain than in the corn-strain.

**Conclusions:**

Together, our results indicate that the allochronic differentiation in the two strains of *S. frugiperda* is associated with differential transcription of *vrille* or a cis-acting gene close to *vrille,* which contributes to the evolution of prezygotic isolation in *S. frugiperda*.

**Electronic supplementary material:**

The online version of this article (doi:10.1186/s12862-017-0911-5) contains supplementary material, which is available to authorized users.

## Background

Virtually all life on earth experiences a similar day-night cycle, yet some species have evolved to be day-active, while others are night-active. Even closely related species living in the same habitat can differ from each other in their daily activity rhythms [1]. The molecular mechanisms of circadian rhythms have been revealed in a broad range of eukaryotic species from algae [2] to mammals (reviewed in SM Reppert and DR Weaver [3]), and also in insects with the increasing number of sequenced insect genomes [4–7]. Surprisingly, despite this growing knowledge on the molecular basis of circadian rhythms, the evolution of differentiation in daily activity patterns is largely unexplored. In insects, differences in diurnal mating times have been found to prevent gene flow between populations [8–14]. By determining the genetic basis of allochronic differentiation between closely related species, or even between divergent populations within species, the initial steps causing differentiation in daily activity rhythms can be discovered, which is important for an understanding of the evolution of circadian rhythms on a micro-evolutionary time-scale.

An ideal model organism for the study of the evolution of circadian rhythms is the noctuid moth *Spodoptera frugiperda* (Lepidoptera: Noctuidae), as it consists of two naturally occurring morphologically identical strains that exhibit strain-specific timing of mating in the night [15, 16]. The so-called corn- and rice-strains seem to be in the process of ecological speciation in sympatry [17]. Although the hybridization rate is up to 16% in the field [18], the two strains do not merge into one panmictic population, which is probably prevented by a combination of different isolation barriers [17]. So far, three possible prezygotic mating barriers have been described in this species: a) differential host plant choice [19–23], b) strain-specific timing of mating in the night [15, 16], and c) female sex pheromone differences [24–26]. Recent studies have shown that host preference in the field is not as specific as previously thought [27–29]. Therefore, habitat isolation seems to be a relatively weak prezygotic mating barrier. Differences in female sex pheromone composition are also likely to constitute a weak prezygotic mating barrier [26, 30]. As both strains consistently differ in their timing of reproductive activity at night [15, 16], allochronic divergence seems to be a major barrier separating the two *S. frugiperda* strains. The corn-strain calls, mates and oviposits approximately three hours earlier than the rice-strain, with only a small overlapping time-window between the strains [15, 16].

Allochronic speciation due to *seasonal* timing differences has been suggested for several insect species, e.g. crickets [31, 32], fruit flies [33, 34] and mosquitoes [13]. However, surprisingly little research has been conducted on the importance and exact genetic changes underlying temporal speciation (reviewed in AT Groot [1]). Recently, a study by Kaiser et al. [35] determined the genomic basis of circadian and circalunar timing adaptations in the midges *Clunio marinus*. Different naturally occurring strains of C*. marinus* emerge at different time points in the circadian as well as the circalunar rhythm and mate and oviposit shortly after. The abundance of different splice variants of the calcium/calmodulin-dependent kinase II.1 (CaMKII.1) is associated with these allochronic differences.

Also the two *S. frugiperda* strains differ in their *diurnal* mating patterns and we hypothesize that genetic and/or expression differences in one or more clock genes underlie their differences in timing of reproductive activity.

**Fig. 1:**
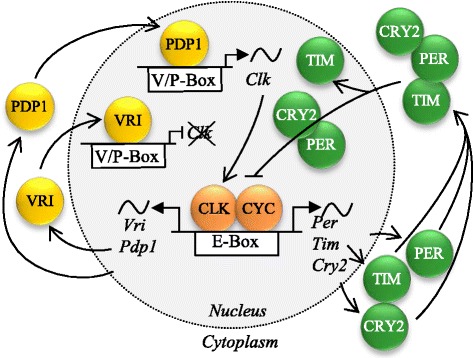
Two feedback loops that define the circadian rhythm in *Danaus plexippus*. Adapted from S Zhan et al. [5]. In the first feedback loop (green proteins), the CLK:CYC dimer promotes the transcription of *Per*, *Tim* and *Cry2*. PER, TIM and CRY2 proteins enter the nucleus where a PER:CRY2 dimer inhibits the binding of the CLK:CYC dimer to E-Boxes and thus inhibits the expression of genes with E-Box promoters. In the second, modulatory feedback loop (yellow proteins), the CLK:CYC dimer promotes the transcription of *Vri* and /or *Pdp1.* VRI and PDP1 proteins enter the nucleus, where PDP1 promotes *Clk* transcription while VRI inhibits *Clk* transcription

In general, biological clocks are a network of genes and gene products that enhance and suppress each other in a rhythmic manner, entrained by environmental factors such as light, temperature or tides [36, 37]. Within insects, the clock gene network is best described in the vinegar fly *Drosophila melanogaster*, where the network consists of two interlocked feedback loops [36, 38]: one involving the genes *vrille* (*vri*), *PAR-domain protein 1* (*pdp1*), *clock* (*clk*) and *cycle* (*cyc*)*;* and the other incorporating *period* (*per*), *timeless* (*tim*), *clk* and *cyc*. In addition, kinases phosphorylate clock proteins (e.g. phosphorylation of PER by DOUBLETIME (DBT) and CASEIN KINASE2α (CK2α)) and facilitate their accumulation [36], while *cryptochrome 1* (*cry1*) functions as a circadian photoreceptor. Most of these genes are also present in Lepidoptera [5, 39–42], and are thus good candidate genes that may underlie the timing differences between the corn- and the rice-strain (see Fig. 1). Additionally, a second cryptochrome, *cryptochrome 2* (*cry2*), is present in Lepidoptera and able to repress CLK: CYC mediated transcription [40, 41, Fig. 1]. Even though most of the clock genes and their organization in the circadian clock are well conserved across taxonomic groups [43, 44], differences among insects orders have been highlighted [41, 43, 45], especially in the daily oscillation patterns of gene expression [46–49]. Identifying the exact genetic changes and the mechanism(s) underlying allochronic differentiation in behavior will shed light on how temporal differentiation may evolve.

In this study, we determined the genetic basis of the major prezygotic isolation barrier, i.e. differentiation in the diurnal mating patterns of the two strains of *S. frugiperda*. To identify the genomic loci (chromosomes) that explain most of the variance in timing of mating activity in an unbiased way, we first conducted quantitative trait locus (QTL) analyses using AFLP markers and RADtags. To determine which of the candidate clock genes map to the major Quantitative trait chromosome (QTC), we indirectly mapped these genes onto our genetic map by homologizing the *S. frugiperda* linkage map to the *B. mori* chromosomes and identifying the location of the candidate genes on the homologous chromosomes. This approach is possible due to the high syntheny between *S. frugiperda* and *B. mori* [50]. Since *vrille* was located on the major QTC, we determined differences in expression levels, as well as sequence differences of this gene, between the two strains, and compared this to differences in the other main clock genes.

## Methods

As *S. frugiperda* is a non-model organism, no assembled genome was available when we started investigating the genetic basis of the differences in mating time of the two strains. Hence, this manuscript presents results that were obtained with a variety of methods to overcome this lack of information. In the course of experiments, the genome of both the corn- and the rice-strain became available to the members of The Fall armyworm International Public Consortium, which facilitated genomic comparisons and expression analysis of all main clock genes. An overview of the chronology of the experiments is depicted in Additional file 1.

### Insects

Individuals used for the QTL analysis descended from > 200 rice-strain larvae and > 100 corn-strain larvae, collected from different fields in Florida in 2003 and 2004, respectively (Additional file 2). These populations were reared for 10 (corn-strain) and 21 (rice-strain) generations in mass culture at the USDA-ARS in Gainesville, FL, before shipment to the Max Planck Institute for Chemical Ecology (MPICE) in 2007. These populations were also used by G Schöfl, DG Heckel and AT Groot [16]. We refer to these populations as CL1 and RL1 (Additional file 2). Unfortunately, these two populations died after six years of laboratory rearing. Therefore, we established new laboratory populations starting with ~ 300 larvae collected in Florida (rice-strain) and Puerto Rico (corn-strain) in 2010 (Additional file 2), which were shipped directly to MPICE, where all adults were screened for strain-specific COI markers [23], and separated accordingly into strain-specific colonies. We refer to these populations as CL2 and RL2 (Additional file 2). All populations were reared in climate chambers with reversed light:dark (L:D) cycle (photophase started at 10 pm, scotophase started at noon) and 14:10 L:D photoperiod at 26 °C and 70% RH. Adults were fed with a 10% honey-water solution and random single-pair-matings were set up to maintain the populations and minimize inbreeding.

### Generation of backcrosses

For the QTL analysis, we generated female-informative backcrosses (Additional file 3). Single pair matings between pure corn- and rice-strain individuals were performed to obtain F_1_ hybrids (denoted CR from corn mothers and RC from rice mothers). Hybrid females were then backcrossed to pure rice-strain males to produce different backcross families (Additional file 3). Two backcross families (BCs) were used for the QTL analysis (BC_A: RCxR, BC_B: CRxR) (the first two letters of a backcross refer to the mother, the last letter to the father). The two rice-strain fathers used to generate both backcrosses were kin.

### Phenotyping backcross families

To determine the phenotype for the QTL analysis, we observed the mating behavior of a) pure strain individuals in intra-strain (CxC, RxR) and inter-strain matings (CxR, RxC), b) hybrid females backcrossed to pure strain males (CRxC, CRxR, RCxC, RCxR), and c) female backcross offspring crossed to pure strain males (CR-RxC, CR-RxR, RC-RxC, RC-RxR). The observations of mating behavior were performed as described by [16] and summarized here. One to four day-old virgin females and males were set up in single pairs in clear plastic cups (16 oz.) and provided with 10% honey solution. All matings were set up simultaneously and placed in a walk-in climate chamber (26 °C, 70% RH, L:D 14:10) two hours before scotophase. In total, 320 to 400 couples were observed throughout the scotophase and one hour into photophase (in total 11 h), with a 30 min interval, i.e. each couple was observed once every 30 min. All pairs were observed for three consecutive nights starting at the first day of the mating. The onset time of the first mating was the phenotype used for the timing QTL analysis. After observation, all individuals were frozen at -80 °C for further genetic analysis.

### Genetic map construction

DNA of 90 randomly chosen backcross females (44x RC-R, 46x CR-R) as well as of their parents and grandparents were used to generate AFLP markers. Female backcross individuals were chosen to construct the genetic map and conduct the QTL analysis, as Schöfl et al. 2009 [16] showed that the onset time of mating was mainly influenced by the female mating partner (i.e. mating time was significantly different between corn-strain and rice-strain females, irrespective of the strain-identity of their mating partner). After DNA extraction, AFLP markers were generated as described in Groot et al. [51], and summarized here: 200 ng DNA of each sample was digested with EcoRI and MseI (New England Biolabs, Ipswich, MA, USA), and EcoRI- and MseI-adapters were ligated to the fragments, preamplified and selectively amplified with different EcoRI- and MseI-primer combinations (Additional file 4). The generated AFLP fragments were analyzed on a 6.5% polyacrylamide gel using a LI-COR 4300 DNA analyzer (LI-COR Biosciences, Lincoln, NE, USA). AFLP gels were scored with AFLP-Quantar Pro 1.0 (KeyGene, Wageningen, The Netherlands). To identify corn-strain specific markers, we scored markers that were present in the corn-strain grandparent (C grandmother or grandfather), the hybrid mother (RC or CR), and half of the offspring females (heterozygote females), but absent in the rice-strain grandparent (R), the backcross male (R), and the homozygote backcross (CR-R and RC-R) females. For identification of rice-strain specific markers, we scored markers present in the rice-strain grandparent, the hybrid mother and the homozygote offspring females, but absent in the corn-strain grandparent, the father and the heterozygote backcross females. All markers were converted to the same phase by inverting the absence/presence patterns of all rice-strain specific markers.

After scoring at least 450 markers, we constructed a linkage map for each BC with MapMaker 3.0 (http://www.broadinstitute.org/ftp/distribution/software/mapmaker3/). Markers were clustered into linkage groups (LG) using a LOD of 4.5. In BC_A, 30 LGs were identified that refer to the 30 autosomes in a backcross family, as there is no crossing over in lepidopteran females [52]. In BC_B, 29 LGs were identified. The chromosome names (chromosome 1 to 30) were chosen arbitrarily for each linkage map, so that the same numbers in the different linkage maps are not necessarily homologous. Markers present in both backcrosses (Additional file 4) were used to homologize the chromosomes of these backcrosses.

### QTL analysis

A QTL analysis based on AFLP markers was conducted with two female-informative backcross families. A total of 465 (in BC family A) and 514 (in BC family B) informative AFLP markers were used to identify the 30 *S. frugiperda* autosomes (Additional file 4). Each chromosome consisted of at least two AFLP markers from different primer combinations up to a maximum of 26 markers. Seventeen markers in each BC family did not map to any linkage group. Three chromosomes (corresponding to three linkage groups in BC_A and 2 linkage groups in BC_B) could not be homologized between the two linkage maps.

To identify candidate QTC, each chromosome was tested for a significant difference in the phenotype (i.e. onset time of first mating) between the homo- and heterozygote backcross females. The two backcrosses were also combined for this analysis, to increase the sample size and thus the possibility to detect QTC. Because of the absence of crossing-over in female Lepidoptera [52], each identified QTC corresponds to an individual chromosome, on average 1/31 of the genome. Statistical analysis was performed with R 2.5.0 (R-Development-Core-Team, 2007) and SAS® software (SAS institute, Cary, NC, USA, 2002-2008). To assess how much of the variance is explained by the different QTC (R^2^ value) we conducted a two-sided *t*-test and a GLM (The GLM procedure of the SAS software, with onset time of the first copulation as dependent variable). Chromosomes with a significant correlation (*P* < 0.05) were considered a QTC.

### Homologizing linkage maps to *Bombyx mori* chromosomes, using RAD markers

To identify candidate genes on the QTC, the linkage map was homologized to the reference genome of *B. mori,* using restriction site associated DNA (RAD) analysis (see Baxter et al [53] and Groot et al. [54]). DNA of parents, female grandparents and 11 backcross individuals per backcross family was digested with the Sbf1 restriction enzyme (New England Biolabs, Ipswich, MA, USA), barcoded, pooled, sheared and amplified, following the procedure described in Groot et al [54]. The pool was paired-end sequenced (50 bp fw, 50 bp rev) by FASTERIS (Geneva, Switzerland) with a HiSeq Illumina sequencer, resulting in 76 million reads. The reads were separated by barcodes into pools per individual and filtered for quality (q10 = 99%). On average, there were 5-10 different paired-end reads per forward read (Additional file 5). The segregation patterns that were obtained with the AFLP markers for different linkage groups were utilized to identify RAD markers segregating in the same pattern with RAD tools [53]. The AFLP markers showed a specific presence (1) /absence (0) pattern in backcross individuals for each chromosome, thus RAD markers showing the same 1/0 pattern in the backcross individuals were identified as belonging to the same specific linkage group. All sequences matching an AFLP segregation pattern were pooled across the individuals, after which the paired-end sequences were retrieved, resulting in 30 FASTA files (one file per chromosome). Each group was assembled into RAD contigs using CLC Genomics Workbench (CLC bio version 5.0.1; www.clcbio.com). Sequences were trimmed for length and quality with standard settings (nucleotide mismatch cost = 2; in/del cost = 2; length fraction = 0.35; similarity = 0.9; when bases conflicted, the base with highest frequency was chosen) and assembled *de novo.* Contig sizes ranged from 89 – 598 bp, with the majority of contigs being 100 – 250 bp long.

Resulting contigs from the paired-end RAD sequences were BLASTed against the scaffolds of the corn-variant assembly 3.0 and the rice-variant assembly 1.0 of the *S. frugiperda* genome [55], http://bipaa.genouest.org/is/lepidodb/spodoptera_frugiperda/. The scaffolds with the best BLAST hits were BLASTed in SilkDB (http://silkworm.genomics.org.cn/) and KAIKObase [56], http://sgp.dna.affrc.go.jp/KAIKObase/]. We considered the *S. frugiperda* chromosomes and *B. mori* chromosomes to be homologous when a) multiple contigs of the same *Sf* chromosome produced significant BLAST hits (e-value < E-10) to the same *Bm* chromosome, or b) in cases where multiple *Bm* chromosomes hit contigs of one *Sf* chromosome, the hit with the lowest e-value was chosen (Additional files 6 and 7).

After homologizing the *S. frugiperda* chromosomes to the *B. mori* chromosomes, we assessed the location of candidate genes involved in the circadian rhythm (Fig. 1), using KAIKObase (http://sgp.dna.affrc.go.jp/KAIKObase) and the homology table (Additional file 6). The position of *vri* on the timing QTC *Sf*_C25 (*Bm*_C27) was verified by mapping it via single nucleotide polymorphisms (SNPs) to the combined linkage map, using the segregation patterns of the SNPs in the grandparents, parents, and 8 backcross females of both backcross families (see Additional file 8).

### Expression analysis of the main candidate gene, *vrille*

To determine strain-specific expression differences and an overview of the daily oscillation in the candidate gene *vri,* as well as the main other circadian clock genes *period, timeless, cryptochrome 2*, PAR-*domain protein 1, clock and cycle* (Fig. 1), we conducted two reverse transcription-quantitative real-time PCR (RT-qPCR) experiments with mRNA from heads of female *S. frugiperda* of both strains. We chose female heads for this experiment, as the mating process is started by female calling, followed by male courtship and then copulation. Thus, the mating time is influenced significantly more by the female partner than by the male partner [16]. In the first experiment, 15 females of both strains were transferred from the rearing cups to a 10 ml Falcon tube, immediately frozen in liquid nitrogen and kept at -80 °C, which was repeated every hour for 24 h. RNA was isolated from three pools of five heads, providing three biological replicates per strain per time point. RNA extraction, cDNA synthesis and RT-qPCR reaction were conducted, as described in AT Groot et al. [54] and summarized here. Pools of five heads were ground with mortar and pestle in liquid nitrogen, RNA was isolated using Direct-zol™ RNA MiniPrep (Zymo Research corp.), and DNase was digested by adding 10 μl 10x Turbo DNase buffer and 1 μl Turbo DNase (Ambion, LIFE TECHNOLOGIES, Darmstadt, Germany). cDNA was synthesized from 1000 ng RNA using Verso cDNA synthesis kit (Thermo Fisher Scientific, Schwerte, Germany). RT-qPCR experiments were conducted with 5 ng cDNA per reaction, 2 technical replicates on each plate, using ABsolute Blue QPCR SYBR Green Low Rox Mix (Thermo Fisher Scientific, Schwerte, Germany) and Applied Biosystems 7500 Real-Time PCR (ThermoFisher Scientific) (see Additional file 8 for details). The Elongation Initiation Factor 1α (eIF1α) was used as the reference gene and amplified for all samples. eIF1α was chosen as the reference gene as it showed the most stable expression over the different time points in a pre-experiment. Relative expression levels were calculated as copy numbers per 1000 copies eIF1α.

To verify our first RT-qPCR results, we conducted a second qPCR experiment where we focused on the 12 most relevant time points in which the two strains showed differences in clock expression levels, i.e. from 5 h before (-5 h) until 6 h into scotophase (+6 h). For this second experiment, we again observed mating couples for one night, as described above, and extracted RNA extraction in the second night, for which we chose corn-strain females that showed reproductive behavior (female calling, copulation) early in the night and rice-strain females that showed reproductive behavior later in the night. Every hour, 6 - 10 females of both strains were transferred from the rearing cups to a 10 ml Falcon tube, immediately frozen in liquid nitrogen and kept at -80 °C. This time, RNA was isolated individually from six heads, providing six biological replicates per strain per time point. RNA extraction, cDNA synthesis and RT-qPCR reaction were conducted on the individual samples. Heads were ground with mortar and pestle in liquid nitrogen, RNA was isolated using innuPREP RNA Mini Kit (Analytik Jena, Germany). RT-qPCR experiments were conducted with 5 ng cDNA per reaction, this time 3 technical replicates on each plate, using 5X Hot firepol EvaGreen® qPCR mix plus (ROX) (Thermo Fisher Scientific, Schwerte, Germany) and a Bio-Rad CFX machine (Biotum) (see Additional file 8 for details). eIF1A was again used as the reference gene and amplified for all samples. Relative expression levels were calculated as above.

Statistical differences in strain-specific expression levels of the clock genes were tested in R (R Development Core Team 2010). For each time point, we tested the normality of the data with a Shapiro-Wilk test (shapiro.test function under R). To ensure that the variances were equal in both rice and corn dataset, we ran a Fisher test (var.test function under R). Finally, we used a Student test to compare the expression levels of two samples for each gene (t.test function in R) with a Bonferroni correction for multiple comparisons.

We also tested for significant difference between the whole expression pattern of the corn-strain and the rice-strain for individual clock genes. For this, we first used a Shapiro-Wilk normality test for each dataset (dataset = expression data 1 gene for one strain for each timepoint) followed by an F test to compare the variances of both strains per clock gene. Finally, we used a Two Sample *t*-test to test for a significant difference between the corn-strain expression and the rice-strain expression for each clock gene.

All graphs were made in R with the ggplot2 package [57].

### Sequence differences of the main candidate gene, *vrille*

To assess strain-specific sequence differences in *vri*, the sequence of the gene was established stepwise. First degenerate primers based on insect ESTs and genomic sequences (gb|AY526608.1, gb|AY576272.1, gb|AADK01019845.1) were used to obtain partial sequences. After obtaining the sequences, primers were designed to sequence further. The DNA Walking SpeedUp™ Kit II (SEEGENE, Eschborn, Germany) was used to obtain the sequence upstream of the coding sequence (see Additional file 9 for all primers used). To determine exon/intron structure, the coding region was sequenced from cDNA. Subsequently, parts of the gene were sequenced in 88 different samples (including backcross individuals and corn- and rice-strain individuals from different regions; Additional file 2), using Sanger-sequencing and Sequencher 4.10.1 for analysis. All obtained sequences are available in GenBank (accession numbers KM675483 - KM675658). Subsequently, the *S. frugiperda* genome for both strains (http://www6.inra.fr/lepidodb/SfruDB) and an in-house RNAseq database of larval guts became available [55]. With the full length mRNA acquired from the RNAseq database and BLASTed against the genome, the full sequence of *vri* was obtained, including a large intron in the 5’UTR. The corn-strain genome was not complete in this region, thus two BAC clones (AUA0AAA25YL06FM1, AUA0AAA20YH15RM1) spanning the region were obtained from the Centre National de Ressources Génomiques Végétales (CNRGV, Toulouse, France) and shotgun sequenced using Sanger sequencing and Sequencher for analysis. Based on the alignment of the rice-strain genome from SfruDB and the BAC clone sequences, additional parts of the intron were sequenced in 12 corn-strain and 12 rice-strain individuals from the CL_1 and RL_1 populations, as well as the parental and F_1_ generations of the backcross families.

When the genome of *S. frugiperda* became available, the other main clock genes were annotated in both strains as part of the WGS project [55]. The coding sequences of each gene of both strains were aligned to identify polymorphisms between the strains. The protein domains were identified on http://prosite.expasy.org/ and using Protein BLAST on http://blast.ncbi.nlm.nih.gov/.

## Results

### QTL analysis

**Fig. 2:**
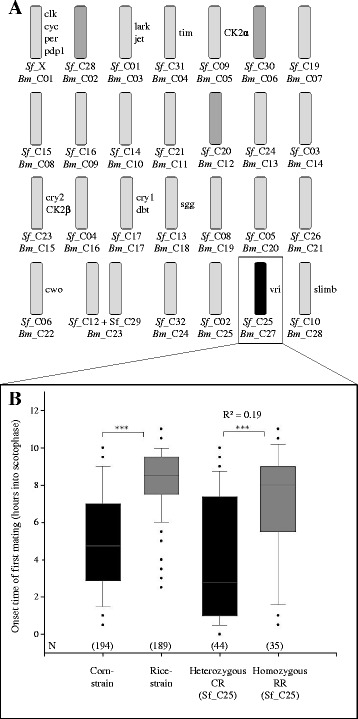
**a** Location of clock-related genes on the homologous chromosomes of *S. frugiperda* and *B. mori*. Chromosomes that do not show a QTL are depicted light grey, minor QTL chromosomes dark grey and the major QTL chromosome black. Names of clock-related genes are positioned on the right of the chromosome they are situated on in B. mori. **b** Phenotype (onset time of first mating) for major QTL chromosome *Sf*_C25 (=*Bm*_C27) of pure corn-strain and rice-strain individuals vs. heterozygous CR and homozygous RR backcross individuals

QTCs were identified by testing each linkage group for a significant association with the phenotypic trait, i.e. onset time of first mating. Resulting *P*-values and *R* ^2^-values (see Additional file 6) refers to the combined analysis of both BCs to use the biggest sample size possible. One QTC (*Sf_*C25, *P* < 0.0001, *R* ^2^ = 0.19) had a major effect on the variance in the strain-specific timing of mating. This QTC is homologous to *Bombyx mori* chromosome 27 (*Bm*_C27) and explained 19% of the variance of the onset time of first mating (Fig. 2). *Bm*_C27 is 14.5 Mb in size (52.8 cM) and represents 3.3% of the total *B. mori* genome [56, 58]. The difference in the onset time of mating between heterozygous and homozygous individuals (carrying only rice-strain copies of the chromosome) for Sf_C27 did not differ between the combined or individual analysis of backcrosses (Additional file 10). The LOD scores of all linkage groups are shown in Additional File 11.

Even though the major QTC *Sf_*C25 explained 19% of the variance between the strains, it only achieved a power of 0.55, i.e. only 55% of QTCs of this magnitude could be detected with our setup on average (Additional file 12). This is due to the small sample size and implies a chance of missing or underestimating minor QTCs. However, the finding of the same major QTC in two independent backcrosses strengthens our finding. We did detect three minor QTCs in the combined analysis that were not consistently present in both families when analyzed individually: *Sf*_C28 (*Bm*_C2, *P* = 0.014, *R* ^2^ = 0.08), *Sf*_C30 (*Bm*_C6, *P* = 0.0104, *R* ^2^ = 0.08) and *Sf*_C20 (*Bm*_C12, *P* = 0.023, R^2^ = 0.07).

### Mapping the candidate genes

The main circadian rhythm genes are located on the following chromosomes (Additional file 6): *per, clk, cyc* and *PdP1* on the sex chromosome (*Bm*_C01), *jetlag* on *Bm*_C3 (*Sf*_C10), *tim* on *Bm*_C4 (*Sf*_C31), *CK2α* on *Bm*_C5 (*Sf*_C09), *cry2* on *Bm*_C15 (*Sf*_C23), *CK2β* and *cry1* on *Bm*_C15 (*Sf*_C23), *dbt* on *Bm*_C17 (*Sf*_C17), *shaggy* on *Bm*_C18 (*Sf*_C13), *clockwork orange* on *Bm*_C22 (*Sf*_C6), *slimb* on *Bm*_C24 (*Sf*_C12, 32), *vri* on *Bm*_C27 (*Sf*_C25) and *CK1α* on *Bm*_scaf256 (which has not been mapped to a *B. mori* chromosome, and thus cannot be homologized). Thus, of all candidate genes, only *vri* mapped to the one major QTC *Bm*_C27 (*Sf*_C25). Since the reciprocal F1 hybrids (CR and RC) did not differ in their onset time of mating (Additional file 13; [16]), the involvement of the sex chromosome in the timing differentiation between the two strains can be excluded, which thus excludes *per, clk, cyc* and *PdP1,* which are located on the sex chromosome.

### Expression differences of the main candidate gene *vrille*

**Fig. 3:**
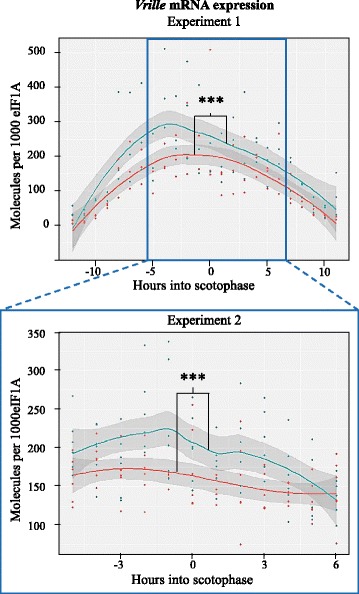
Expression levels of *vrille* for the two strains of *S. frugiperda* (corn-strain in red and rice-strain in blue). The dots represent individual measurements; the line connects the means for each time point and each strain; the grey shaded area represents the confidence interval. Experiment 1 was conducted over 24 h, experiment 2 over 12 time points. Significant difference between the overall expression pattern of the corn-strain and the rice-strain is indicated by the asterisks

The main candidate gene *vrille* showed a clear daily oscillation pattern in its expression in both strains and the overall expression of *vrille* differed between the strains (Fig. 3). We found significant differences in expression levels between the two strains in the first 24 h experiment (experiment 1, *P* = 0.00065), as well as in the second 12 h experiment (experiment 2, *P* <0.0001) (Fig. 3). These differences were due to overall significantly higher expression levels of *vri* in the rice-strain compared to the corn-strain.

**Fig. 4:**
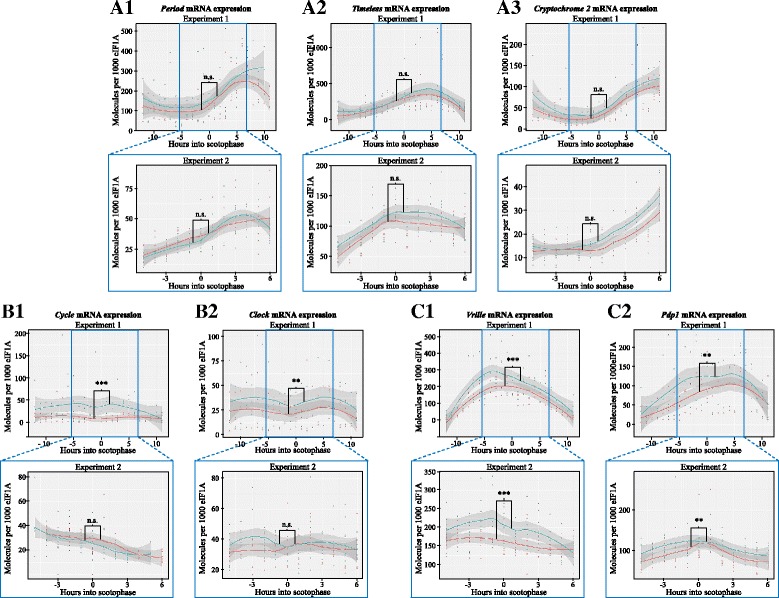
Expression levels of key clock genes for the two strains of *S. frugiperda* (corn-strain in red and rice-strain in blue). The dots represent individual measurements; the line connects the means for each time point and each strain; the grey shaded area represents the confidence interval. Experiment 1 was conducted over 24 h, experiment 2 over 12 time points. Significant difference between the overall expression pattern of the corn-strain and the rice-strain is indicated by the asterisks. A1-A3. Genes of the first, main feedback loop (*period*, *timeless*, *cryptochrome 2*); B1-B2: Core clock genes connecting both feedback loops (*cycle*, *clock*); C1-C2. Genes of the second, modulatory feedback loop (*vrille*, *PAR-domain protein 1;* Fig. 4 = C1 and was repeated to facilitate an overview of the key clock genes)

In comparison, the other clock genes showed rhythmic expression levels as well, except for *clk* and *cyc* which did not show a clear daily oscillation pattern (Fig. 4a-c). Three clock genes, *per*, *tim* and *cry2* did not differ between the strains in either experiment (Fig. 4a1-6a3). While in the first 24 h experiment three additional clock genes did show significant differences between the two strains (*pdp1 P* = 0.00543, *clk P* = 0.00222, and *cyc P* = 0.00002; Fig. 4b, c), only *pdp1* also showed significant differences in the second 12 h experiment (*P* = 0.0038). All differences between the two strains were due to significantly higher expression levels in the rice-strain than in the corn-strain (Figs. 3 and 4).

### Sequence differences of the candidate gene *vrille*

*Vrille* is a short gene without introns in the protein coding region, coding for a 367 aa protein, followed by a 1234 bp 3’ UTR. The 5’ UTR is divided into a 45 bp segment and a 375 bp segment by an intron that contains regulatory elements, namely 11 Ebox elements (Ebox A – Ebox K) with the core sequence CACGTG (Fig. 3). Near Eboxes E, F and I, five strain-specific polymorphisms were identified in the investigated corn-strain and rice-strain populations and the maternal grandmothers of BC_A and BC_B (Table 1).

**Table 1:** Variation in the regulatory intron in the 5’UTR of *vri*

Position	Close to	Type	Population		Individual	
			CL_1 Corn	RL_1 Rice	mgmA Rice	mgmB Corn
4717	EboxE	SNP	T	A	A	T
4727	EboxE	SNP	T	A	A	T
4816	EboxE	IN/DEL	_ _ _ _ _ _	TTCGAA	TTCGAA	_ _ _ _ _ _
4870	EboxF	SNP	A	C	C	A
6690	EboxI	SNP	T	A	A	n.a.

Single nucleotide polymorphisms (SNPs) and insertions/deletion (IN/DEL) between 12 individuals from a corn-strain population (CL_1) and 12 individuals from a rice-strain population (RL_1) as well as in the maternal grandmothers (mgm) of BC_A and B (originating from these populations). Mgm = maternal grandmother; Sample name followed by Corn (= corn-strain) or Rice (= rice-strain); n.a. not available due to sequencing restrictions

The availability of a corn-strain and a rice-strain variant of the *S. frugiperda* genome assembly enabled us to compare the sequences of the main clock genes in these assemblies (see Additional file 14). Sequence differences of the main clock genes were assessed by identifying non-synonymous as well as synonymous SNPs in the protein coding regions. We mostly found synonymous (syn) SNPs between the corn-strain and the rice-strain genome assembly, with the exception of clk, cyc and tim, which showed two, two and one non-synonymous (non-syn) SNPs, respectively (Table 2). All non-synonymous SNPs were located in non-conserved domains of these genes (Additional file 14).

**Table 2:** Number of SNPs between the corn-strain and rice-strain variant of the *S. frugiperda* genome assembly

Gene name	Amino acids	Syn SNPs total	Syn SNPs per aa	Non-syn SNPs total	Non-syn SNPs per aa
*pdp1*	263	3	0.0114	0	0.0000
*dbt*	346	1	0.0029	0	0.0000
*vri*	367	17	0.0463	0	0.0000
*cry1*	528	50	0.0947	0	0.0000
*cry2*	793	23	0.0290	0	0.0000
*per*	1222	34	0.0278	0	0.0000
*clk*	614	29	0.0472	2	0.0033
*cyc*	681	55	0.0808	2	0.0029
*tim*	1279	28	0.0219	1	0.0008

## Discussion

In this study, we aimed to identify the genetic basis of the main prezygotic isolation barrier between the two strains of *S. frugiperda*, i.e. allochronic differentiation. We found one consistent Quantitative trait chromosome (QTC) that significantly accounted for the difference in the onset time of mating in the two strains, Sf_C25, which is homologous to Bm_C27. Detecting only one major QTC is of note because the timing of behavior is a complex trait, depending on the complex network of the circadian clock and its interlocked feedback loops of transcription and translation (see Fig. 1). Thus, one might anticipate that the difference in mating time between the corn and rice strains would represent a polygenic trait affected by multiple loci of modest to small effect. Yet, differences in one or more genes on *Sf*_C25 explain 19% of the timing variance observed between the strains, i.e. most likely there are more loci affecting this phenotype that we did not detect in this study. QTC *Sf*_C25 is a single autosome and the homologous chromosome in *B. mori* (*Bm*_C27) is 14.5 Mb (52.8 cM). This size is comparable to QTL intervals found in other QTL studies [59–62]. In *B. mori* only one candidate clock gene is known to be located on this chromosome, namely *vri*. All other known clock genes map to different chromosomes in Lepidoptera.

The major QTC *Sf_*C25 explained 19% of the variance between the strains, while three minor QTCs in the combined analysis were not consistently present in both families when analyzed individually. In addition, no known genes involved in the circadian rhythm are located on the *Bombyx* homologs of these minor QTCs. Possibly, allochronic differentiation between the two strains is affected by an interaction between different factors involved in the circadian rhythm regulation.

A limitation of our indirect mapping approach is the different number of autosomes in *B. mori* (27) and *S. frugiperda* (30). When homologizing *Helicoverpa armigera* and *B. mori*, Sahara et al. [63] found *Bm* chromosomes 11, 23 and 24 to be merged from two chromosomes each. For *Bm* chromosome 23, we identified two homologous chromosomes in *S. frugiperda*: *Sf*_C12 and *Sf*_C29. Whether Bm_C11 and Bm_C24 are also represented by two chromosomes in *S. frugiperda* remains to be determined. The incomplete homology did not affect our result, because a) we confirmed the position of *vrille* on our major *Sf* QTC, b) all minor QTC have a confident homologue in *B. mori* (see Fig. 2, Additional file 6), none of which contain known clock genes, and c) we homologized all chromosomes with known clock genes. The high synteny level between *B. mori* and *S. frugiperda* [50] also supports our conclusion that *vrille* is the only clock gene located on a QTC in *S. frugiperda*.

### Candidate gene *vrille*

Within the network of the circadian clock genes in insects, *vri* is a powerful player (e.g. in fire ants [64], pea aphids [65] and bean bugs [66]) and best described in *Drosophila* [36, 38, 67, 68]. VRI inhibits *clk* transcription, and since a dimer of CLK and CYC promotes many E-Box promoted genes, *clk* inhibition represses transcription of the core clock genes. Consequently, *vri* mutants have altered behavioral rhythms [68]. Hence, in *S. frugiperda* a strain-specific difference in *vri* structure or expression may cause a strain-specific expression difference in other clock genes, leading to a timing difference in behavior.

Our qPCR results show that *vri* is consistently higher expressed in the rice-strain compared to the corn-strain (Fig. 3). Since VRI inhibits the transcription of *clk,* which is needed for the expression of most circadian rhythm genes, differences in *vri* expression may impact the expression of the downstream players of the interlocked feedback loops. Interestingly, we only found strain-specific expression differences in the genes that constitute the second, modulatory feedback loop: *vri*, *pdp1*, *clk* and *cyc* with the rice-strain showing an overall higher expression of these genes in at least one of the experiments. *Pdp1*, *clk* and *cyc* are all located on the sex chromosome, which is not involved in the timing differentiation between the strains (see Additional file 13). Thus, the observed expression pattern differences reflect downstream temporal regulation dependent upon the initial upstream genetic difference in the expression of *vrille*. The fact that only one feedback loop shows expression differences between the strains is surprising, as the CLK:CYC dimer connects and promotes the transcription of the downstream genes in both feedback loops (Fig. 1). Yet, mRNA expression differences do not directly translate to differences at the protein level. For the generally low turnover of the CLK and CYC proteins, the total effect of mRNA expression differences on protein abundance can be subtle. Additionally, there is always competition for the CLK:CYC dimers between the E-Box promoter genes, and basic helix-loop-helix transcription factors like CLK and CYC exhibit differences in their binding specificities to E-Box binding sites [69]. Since *per* and *tim* showed higher expression peaks compared to *pdp1* and *vri*, it is possible that *per*- and *tim* E-Boxes attract CLK:CYC dimers more easily and are thus less affected by a limited CLK:CYC dimer abundance.

Differences in expression levels of clock genes have been shown to be linked to different activity patterns. For example, the migratory songbird *Emberiza melanocephala* shows significant differences in clock gene expression levels, rather than phase shifts, between life history states that differ in their daily activity patterns (e.g. night activity in the migratory life state and day activity in the pre-migratory state) [70]. In insects, the cricket *Gryllus bimaculatus* shows lower expression of *tim* and *per* in the nocturnal nymphs compared to the diurnal adults [71]. Whether and how the differences in transcription levels of clock genes in *S. frugiperda* strains may affect behavioral variation in timing of sexual activities remains to be determined.

**Fig. 5:**
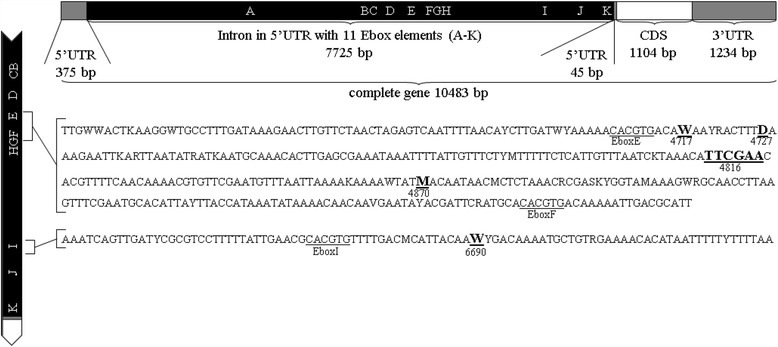
Structure of *vri* in the corn- and rice-strain of *S. frugiperda* and strain-specific polymorphisms in the intron in the 5’UTR

In our search for sequence differences in *vri* that might account for the timing difference, we found five strain-specific polymorphisms surrounding E-boxes between the corn-strain and a rice-strain population from Florida and in the parents of the backcross families used for the QTL analysis (Fig. 5). Since the binding specificity of basic helix-loop-helix transcription factors, such as CLK and CYC, is influenced by the genomic region surrounding the E-box binding site [69], a less efficient binding of a transcription factor to the active *vri* E-Box element (s) in e.g. the corn-strain could change the expression of *vri*. Alternatively, a cis-regulatory element regulating this gene could be situated on the same chromosome at a more distant region that we did not yet sequence. Mutations in cis-regulatory elements generally cause expression differences [72, 73] and are hypothesized to be key elements of evolutionary changes [74].

When aligning the protein coding regions of the other annotated clock genes in the corn-strain and rice-strain variant of the *S. frugiperda* genome, we identified a number of synonymous SNPs in every clock gene, and five non-synonymous SNP in *clk*, *cyc* and *tim*. However, none of these non-synonymous SNPs affected conserved protein domains of these genes. Also, these SNPs are based solely on the genome alignments and were not confirmed by testing multiple corn- and rice-strain individuals and may thus not be strain-specific. *Tim* is located on *Sf*_C31 (*Bm*_C04), which is not a QTC for the timing differences. Also, *tim* did not exhibit any strain-specific expression differences (Fig. 4a). *Clk* and *cyc* are situated on the sex-chromosome, which does not underlie the strain-specific timing differences, based on the observation of reciprocal backcrosses. The expression differences of these genes can be explained as a downstream-effect of differences in the expression of *vri* and *pdp1*.

### Comparisons of clock gene expression patterns between species

To compare the expression patterns of the main clock genes in the night-active Lepidoptera *S. frugiperda* to expression data in other species is difficult, as expression studies addressing the clock genes are often conducted under Zeitgeber time (ZT) conditions (L:D 12:12), while we used a longer day (L:D 14:10). With this difference in mind, some expression levels can be compared.

In the day-active migratory Lepidoptera *Danaus plexippus,* and in the day-active *Drosophila melanogaster, vri* expression is highest early in the scotophase [38, 68]. In S*. frugiperda*, *vri* peaks earlier, namely 4 h before the scotophase in the rice-strain and 3 – 1 h before the scotophase in the corn-strain (24 h experiment)*.* In *S. frugiperda, pdp1* shows an expression peak 8 h after *vri* in the rice-strain and 4-7 h after *vri* in the corn-strain. In *Drosophila* expression levels of *pdp1* peaked 6 h after *vri* [38] and thus more comparable to the rice-strain. These different times of peak expression levels are expected, as VRI and PDP1 proteins both bind to the promoter region of *clock* at different time points to inhibit or facilitate *clock* expression, respectively.

As for other clock genes, in both *D. plexippus* and *D. melanogaster, tim* expression peaks 2 h into scotophase (ZT 14) [75], while we found the highest expression levels of *tim* at 4 h into scotophase. In addition, in *D. plexippus*, *per* expression peaks 2 h into scotophase (ZT 14) [75], while we found the highest expression levels of *per* at 6-7 h (corn-strain) to 10 h into scotophase (rice-strain; 24 h experiment). Since TIM, PER and CRY2 proteins form a trimer to enter the nucleus, similar expression patterns are expected in these genes, so that the peak differences between *per* and *tim* in *S. frugiperda* is surprising.

Expression differences in clock genes are especially interesting in the context of diurnal and nocturnal activity patterns. Martin-Fairey et al. [76] investigated PER protein expression levels in diurnal grass rats *Arvicanthis niloticus*. When comparing individuals that exhibited the usual day-active behavior to individuals that had adopted a night-active behavior, they found PER protein expression levels comparable to nocturnal and diurnal rodents [76]: The expression of PER protein peaks in the early morning in diurnal grass rats [76, 77] while it peaks in the late night in nocturnal rodent species [78, 79]. To be able to make such comparisons in Lepidoptera, clock gene expression studies across species should be conducted under comparable conditions.

## Conclusion

In summary, we identified one major QTC for the timing difference in mating between the two *S. frugiperda* strains. The clock gene *vrille* (*vri*) is located on this QTC and thus the major candidate for the strain-specific timing differences. Strain-specific expression differences, as well as strain-specific polymorphisms in the regulatory region of *vri,* support the hypothesis that *vri* plays a key role in the timing differentiation of these two strains. As allochronic, diurnal differentiation is likely the major isolation barrier driving divergence between *S. frugiperda* populations, the mechanism by which *vri* and the other circadian clock genes influence this differentiation should be elucidated, which is possible through fine-scale mapping and functional analyses. This will advance our understanding of the molecular basis of incipient speciation in sympatry.

## Additional files

Additional file 1: Chronological outline of experiments. (PDF 86 kb)
Additional file 2:
*Spodoptera frugiperda* populations. (PDF 68 kb)
Additional file 3: Generation of female-informative backcross families for QTL analysis. (PDF 61 kb)
Additional file 4: Numbers of scored AFLP markers. (PDF 56 kb)
Additional file 5: Coverage of RAD sequences. (PDF 224 kb)
Additional file 6: Homologous chromosomes of *S. frugiperda* and *B. mori*. (PDF 20 kb)
Additional file 7: Details of BLAST hits of *Sf* BC contigs to scaffolds of *Sf* genome and of BLAST hits of *Sf* genome scaffolds to *Bm* genome. (PDF 57 kb)
Additional file 8: PCR conditions used in the mentioned experiments. (PDF 47 kb)
Additional file 9: Primer combinations and annealing temperatures (T_a_). (PDF 71 kb)
Additional file 10: Mean onset time of mating for individuals that are homozygous or heterozygous for the QTC *Sf*_C25. (PDF 107 kb)
Additional file 11: LOD scores for all linkage groups. (PDF 36 kb)
Additional file 12: Power analysis for backcross families. (PDF 317 kb)
Additional file 13: Mating time in *S. frugiperda* hybrids. (PDF 29 kb)
Additional file 14: Position of SNPs between the corn-strain and rice-strain of *S. frugiperda* in the coding regions of the major clock genes. (PDF 76 kb)
