# Supplementary material for: Genetic basis of allochronic differentiation in the fall armyworm
Source: BMC Evol Biol. 2017 Mar 6;17:68. doi: 10.1186/s12862-017-0911-5 (PMC5339952; doi:10.1186/s12862-017-0911-5)
Supplement: Additional file 1: — Chronological outline of experiments. (PDF 86 kb) [file 12862_2017_911_MOESM1_ESM.pdf]

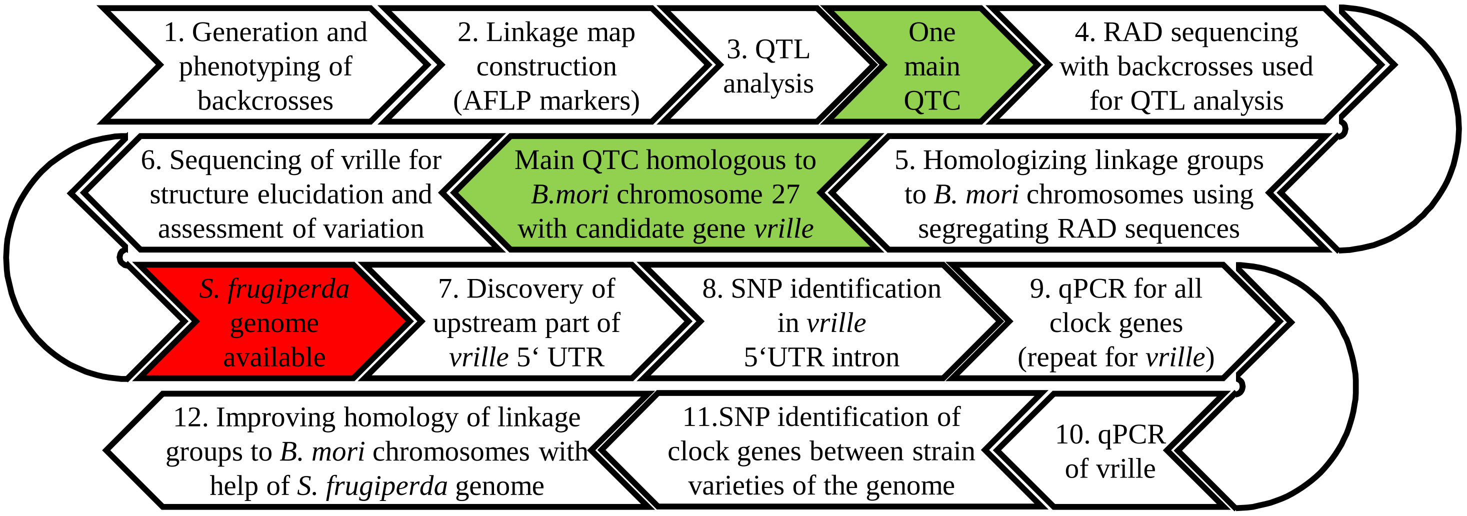

Additional file 1

Chronological outline of experiments. Experiments are shown in white arrows;

Results leading to setup of follow-up experiments are shown in green arrows;

Availability of *S. frugiperda* genome is shown in red arrow.
