# Supplementary material for: Genetic basis of allochronic differentiation in the fall armyworm
Source: BMC Evol Biol. 2017 Mar 6;17:68. doi: 10.1186/s12862-017-0911-5 (PMC5339952; doi:10.1186/s12862-017-0911-5)
Supplement: Additional file 2: — Spodoptera frugiperda populations. (PDF 68 kb) [file 12862_2017_911_MOESM2_ESM.pdf]

Additional file 2

Spodoptera frugiperda populations.

The origin of the populations used for different experiments

Experiment	Strain	Population Origin		Field	Date	Name
Timing QTL	Corn	Florida	Homestead	Corn	2004	CL1
	Rice	Florida	Ona	Grass	2003	RL1
Genetic analysis of Vrille	Corn	Argentina	Los Pereyra	Corn	2010	CF1
		Argentina	Santo Tomé	Corn	2008	CF2
		Florida	Homestead	Corn	2004	CL1
		Puerto Rico	Santa Isabel	Corn	2010	CL2
	Rice	Argentina	Benjamín Aráoz	Grass	2008	RF1
		Argentina	Berón de Astrada	Rice	2008	RF2
		Paraguay	San Cosme y Damián	Rice	2008	RF3
		Texas	College Station	Corn	2010	CF3
		Florida	Ona	Grass	2003	RL1
		Florida	Moore Haven	Grass	2010	RL2
	Corn & Rice	Florida	Hague	Corn	2011	Pheromone trapping
		Argentina	La Cocha	Corn	2008	CF4
Expression analysis	Corn	Florida	Homestead	Corn	2004	CL1
	Rice	Florida	Ona	Grass	2003	RL1
