# Supplementary material for: Genetic basis of allochronic differentiation in the fall armyworm
Source: BMC Evol Biol. 2017 Mar 6;17:68. doi: 10.1186/s12862-017-0911-5 (PMC5339952; doi:10.1186/s12862-017-0911-5)
Supplement: Additional file 3: — Generation of female-informative backcross families for QTL analysis. (PDF 61 kb) [file 12862_2017_911_MOESM3_ESM.pdf]

Additional file 3
Generation of female-informative backcross families for QTL analysis.

**Backcross Family**	**Female Strain***	**Male Strain***	**Generated Offspring**
A	Rice (33)	Corn (22)	F_1 hybrid (RC)
	RC hybrid (1)	Rice (34)	Backcross (**RC-R**)
B	Corn (22)	Rice (33)	F_1 hybrid (CR)
	CR hybrid (1)	Rice (34)	Backcross (**CR-R**)

*Number in brackets show the generation time of the laboratory populations (Table S1) used for the crosses.
