# Supplementary material for: Genetic basis of allochronic differentiation in the fall armyworm
Source: BMC Evol Biol. 2017 Mar 6;17:68. doi: 10.1186/s12862-017-0911-5 (PMC5339952; doi:10.1186/s12862-017-0911-5)
Supplement: Additional file 4: — Numbers of scored AFLP markers. (PDF 56 kb) [file 12862_2017_911_MOESM4_ESM.pdf]

Additional file 4

AFLP markers

Number of informative AFLP-makers scored per primer combination in the three different backcross families (BC) A-C.)

Primer[1]		Number of AFLP-markers	
**MseI**	**EcoRI**	**BC A**	**BC B**
AAG	AAG	21	18
	ACC	9	5
	ACG	14	11
	ACT	6	8
	CGA	6	7
	CGC	4	5
ACA	AAG	19	17
	ACC	8	11
	ACG	7	8
	ACT	14	16
	CGA	11	11
	CGC	11	13
ACC	AAC	7	10
	ACA	6	9
ACG	AAG	6	9
	ACC	5	3
	ACG	5	2
	ACT	4	5
	CGA	7	7
	CGC	4	3
ACT	AGA	7	20
	AGC	6	8
AGG	AAG	15	10
	ACC	10	11
	ACG	3	2
	ACT	7	6
	CGA	5	5
	CGC	4	2
CAA	AGG	2	4
	ATG	5	9
CAC	CAT	7	15
	TAC	6	9
CAG	GTA	3	3
	TCT	3	3
CAT	AAG	6	10
	ACC	9	11
	ACG	5	6
	ACT	6	3
	CGA	9	12

	CGC	6	3
CCA	ACA	5	11
	TTA	-	3
CCC	GTA	6	7
	TTA	5	5
CCG	AGC	7	6
	TAC	3	8
CCT	AGA	4	4
	AGG	2	2
CGA	AAG	13	8
	ACC	1	8
	ACG	9	6
	ACT	11	9
	CGA	11	5
	CGC	3	3
CTC	AAC	12	12
	CAT	6	10
CTG	AAG	7	9
	ACC	13	15
	ACG	6	7
	ACT	3	2
	CGA	4	5
	CGC	1	3
CTT	AGG	13	15
	ATG	22	21
**Total markers**		**465**[2]	**514**[2]

[1]All primers have a core sequence (MseI- primer: 5'-GATGAGTCCTGAGTAA; EcoRI-primer: 5'-GACTGCGTACCAATTC) plus three selective bases at the end (according to the table).

[2]Of all markers scored in the timing QTL analysis, 294 markers were present in both backcross families A and B.
