# Supplementary material for: Genetic basis of allochronic differentiation in the fall armyworm
Source: BMC Evol Biol. 2017 Mar 6;17:68. doi: 10.1186/s12862-017-0911-5 (PMC5339952; doi:10.1186/s12862-017-0911-5)
Supplement: Additional file 5: — Coverage of RAD sequences. (PDF 224 kb) [file 12862_2017_911_MOESM5_ESM.pdf]

Additional file 5

Coverage of RAD sequences (Distribution of RAD sequences per individual sample

Individual	Average FW read Stackhight	Standard-deviation	Standard-error	Max FW read Stackhight	Min FW read Stackhight	Median FW read Stackhight	Number of different FW reads	Average Copy of PE reads	Standard-deviation	Standard-error	Max PE reads	Min PE reads	Median PE reads	Average Different PE read per FW read	Median Different PE read per FW read
mgmA	426.6307	1018.028	15.83725	32219	2	243	4132	90.00413	111.4997	1.734577	979	1	56.25287	7.683204	3
mgfA	458.7566	2385.873	36.01754	107630	2	225	4388	86.1971	106.1036	1.601758	829	1	56.5	7.999544	3
moA	431.8876	1204.144	18.66273	40573	2	241	4163	85.79489	103.4392	1.603179	884	1	58	7.895268	3
faA	415.4024	1197.019	17.18288	41120	2	224	4853	87.56805	107.9381	1.549422	882	1	55.5	7.377911	3
bcA02	387.7372	1056.735	15.08236	38044	2	210	4909	88.96191	107.6816	1.536898	864	1	59.75	7.242819	3
bcA08	413.1341	1047.458	15.58173	38200	2	228	4519	88.63931	110.1302	1.638269	898	1	57	7.6289	3
bcA18	409.1944	974.1887	13.85632	35374	2	230	4943	87.96394	108.8721	1.548536	855	1	58.5	7.60085	3
bcA24	364.366	1146.624	15.83548	45840	2	189	5243	83.94609	104.3151	1.440646	847	1	54.8	6.664124	2
bcA25	393.1814	1075.532	15.52882	35691	2	217	4797	83.85207	105.0766	1.517124	849	1	53.5	8.147801	3
bcA26	421.4899	1251.074	16.42882	40588	2	195	5799	73.1337	95.20368	1.250193	817	1	43.57143	8.608036	3
bcA39	436.8314	1237.587	16.08613	56709	2	205	5919	79.68049	102.0066	1.32588	1170	1	51.36364	7.618855	3
bcA40	387.1765	929.5574	13.85856	29871	2	212	4499	85.72081	103.8102	1.547682	824	1	58.5	7.287842	3
bcA57	426.8583	1106.231	15.43144	42410	2	223	5139	87.98172	114.7766	1.601084	905	1	51	8.52014	3
bcA60	450.5923	1951.403	25.8856	92590	2	201	5683	82.85137	108.4086	1.438054	951	1	46.4	8.675875	3
bcA71	411.6647	1056.592	14.80832	45594	2	221	5091	92.14743	116.1186	1.627422	900	1	57.25	7.097623	3
mgmB	439.8348	1749.672	25.00552	65713	2	190	4896	84.30461	108.096	1.54486	881	1	51	8.24857	3
mgfB	374.7253	1247.66	19.53048	49200	2	204	4081	87.46333	109.3928	1.712401	789	1	54.8	6.598873	2
moB	415.3913	1221.099	16.44736	43634	2	214	5512	82.76193	107.7499	1.451316	881	1	50.5	8.097242	3
faB	319.5755	529.0829	14.25277	8100	2	222	1378	110.9249	133.7814	3.603886	962	1	73.83333	5.197388	2
bcB62	420.5553	1406.466	17.20455	54590	2	177	6683	72.77307	98.2771	1.202172	860	1	37	8.438276	3
bcB49	420.7763	1953.068	25.45483	109348	2	167	5887	74.83013	94.73558	1.234713	853	1	46.5	7.651266	3
bcB47	457.8441	2020.614	25.69915	106689	2	196	6182	80.17825	105.2327	1.338401	972	1	46.21324	8.549822	3
bcB43	427.5193	1501.815	19.71979	69494	2	205	5800	79.48143	100.2758	1.316685	1093	1	51.0625	8.03931	3
bcB32	460.2086	2572.537	33.6229	136510	2	191	5854	74.8997	98.23262	1.283895	826	1	41	8.406218	3
bcB26	513.6885	2049.415	22.44393	98066	2	115	8338	63.37613	89.00555	0.974734	884	1	17.84659	10.40621	3
bcB25	408.9109	1531.764	20.82732	66322	2	189	5409	80.31784	101.9278	1.385907	780	1	48.6875	8.137549	3
bcB20	468.9908	1946.305	23.00301	99304	2	173	7159	71.97056	96.79316	1.14398	941	1	37.375	8.692136	3
bcB09	430.441	1186.454	16.18009	43249	2	224	5377	84.28062	107.3992	1.464641	998	1	51	8.310954	3
bcB08	393.7708	1149.8	14.46433	38380	2	154	6319	76.13915	105.1656	1.322969	843	1	38	7.464947	2
bcB05	403.9736	2023.704	26.74837	110009	2	190	5724	81.34371	99.26521	1.312041	810	1	52.59839	6.885744	3
