# Supplementary material for: Genetic basis of allochronic differentiation in the fall armyworm
Source: BMC Evol Biol. 2017 Mar 6;17:68. doi: 10.1186/s12862-017-0911-5 (PMC5339952; doi:10.1186/s12862-017-0911-5)
Supplement: Additional file 6: — Homologous chromosomes of S. frugiperda and B. mori. (PDF 20 kb) [file 12862_2017_911_MOESM6_ESM.pdf]

Additional file 6

Homologous chromosomes of *S. frugiperda* and *B. mori*.

Homology is indicated by framed numbers at the intersection of *Bm* and Sf chromosomes. Numbers at intersections correspond to numbers of individual *Sf* contigs that produced significant BLAST hits against the *Bm* chromosome. BLAST hits originating from both BCs were given preference. When no *Bm* chromosome was hit by contigs from both BCs, the highest number of individual hits was given preference. Orange field = BLAST hits produced by contigs originating from BC_A. Blue field = BLAST hits produced by contigs originating from BC_B. Green fields = BLAST hits produced by contigs originating from BC_A+B. Clock genes mapping to the *Bm* chromosomes are indicated in the second column. R^2 and P-values of the *Sf* QTC are given in the two right columns.

Bm chr.	clock genes mapping to *Bm* chromosomes	*Sf chr.*		*Bm chromosomes*																												R² BC_A +BC_B	P BC_A +BC_B
		A	B	1	2	3	4	5	6	7	8	9	10	11*	12	13	14	15	16	17	18	19	20	21	22	23*	24*	25	26	27	28		
1	clk, cyc, per, pdp1	X	X																														
2		28	29		4					1									1													0.077	0.014
3	lark, jet	1	23			11			4	1					3			1	3	1		1		2			2	3			4		
4	tim	31	15				15																		1		4				1		
5	CK2α	9	9					17		1			1						1						1				1	1			
6		30	10						11	2																						0.082	0.01
7		19	25							8																							
8		15	1							2	6						4			1					1		1			2			
9		16	3									6													3		1	0,5			0,5		
10		14	16							1		1	13					1								1	1			1			
11*		21	22											7												1							
12		20	21			1	1		2				2		14			1		2		1			1							0.065	0.023
13		24	24							1				1		1																	
14		3	18				1										3				2					1	1	1					
15	cry2, CK2β	23	7															17					1					1			1		
16		4	2																6	1						1							
17	cry1, dbt	17	20	2			3		2	1		1						5	4	16	1				1	2					2		
18	sgg	13	14							1											4					1					1		
19		8	8					2									1			1	1	13			1	1	1			1	1		
20		5	5			2		1	2		6			1				2	2				9			1							
21		26	12			1			1				1	2						1		2		8									
22	cwo, CaMKII	6	6								4										1				8								
23*		29	28			1							1	2								1				12					2		
23*		12	13																							2							
24*		32																									2						
25		2	17				1					1					4	3										19			1		
27	vri	25	27		1		1														2						1			7		0.191	0.0001
28	slimb	10	11	2		1		1		1	1			1							1										6		
		11																										0,5			0,5		
		18	30									1		1													1						
		22										1						1	1						1	1	1		1				
			4											2	1																		
			19				1													1							0,5	0,5	1,5		0,5		
