# Supplementary material for: Genetic basis of allochronic differentiation in the fall armyworm
Source: BMC Evol Biol. 2017 Mar 6;17:68. doi: 10.1186/s12862-017-0911-5 (PMC5339952; doi:10.1186/s12862-017-0911-5)
Supplement: Additional file 7: — Details of BLAST hits of Sf BC contigs to scaffolds of Sf genome and of BLAST hits of Sf genome scaffolds to Bm genome. (PDF 57 kb) [file 12862_2017_911_MOESM7_ESM.pdf]

Additional file 7

Details of BLAST hits of *Sf* BC contigs to scaffolds of *Sf* genome and of BLAST hits of *Sf* genome scaffolds to *Bm* genome. Salmon shaded first field indicates a contig from backcross A; light-blue shaded first field indicates a contig from backcross B.

BC18-Homolog	Bm Chr	hits	RAD contig	Spodoptera scaffold	E-value To BmChr	% identity (RAD contigs to Sf scaffolds)	alignment length (RAD contigs to Spodoptera scaffolds)	e-value (RAD contigs to Spodoptera scaffolds)
BC18-c01	3	2	BC40q10_c23trans_contig1	scaffold_1049	2E-169			
BC18-c01	3	2	BC18q10_c01trans_contig1	scaffold_1049	2E-169			3.00E-95
BC18-c01	3	2	BC40q10_c23trans_contig7	scaffold_10803	4E-28			
BC18-c01	3		BC40q10_c23_contig3	scaffold_1654	1E-39	93.0	71	2.00E-21
BC18-c01	3	2	BC40q10_c23_contig6	scaffold_1654	1E-39	95.1	101	1.00E-37
BC18-c01	3	2	BC18q10_c01trans_contig1	scaffold_219	0	99.5	195	6.00E-97
BC18-c01	3	2	BC40q10_c23trans_contig1	scaffold_219	0	99.5	195	6.00E-97
BC18-c01	3		BC40q10_c23_contig9	SFRU_RICE_015293	7E-95	100.0	208	7.00E-106
BC18-c01	3	2	BC40q10_c23trans_contig7	superscaffold_1050	0	93.8	194	5.00E-78
BC18-c01	3		BC18q10_c01_contig1	superscaffold_226	1E-36	94.3	175	6.00E-69
BC18-c01	3		BC40q10_c23trans_contig3	superscaffold_226	1E-36	91.2	125	2.00E-40
BC18-c01	6	2	BC40q10_c23_contig10	scaffold_11443	2E-25			
BC18-c01	6	2	BC40q10_c23_contig10	scaffold_5988	2E-36	99.2	129	2.00E-60
BC18-c01	6		BC40q10_c23_contig12	scaffold_5988	2E-36	92.3	78	1.00E-23
BC18-c01	6		BC40q10_c23_contig13	scaffold_5988	2E-36	96.3	108	1.00E-43
BC18-c01	7		BC40q10_c23trans_contig6	scaffold_230	1E-35	99.2	125	3.00E-58
BC18-c01	12	2	BC40q10_c23trans_contig10	scaffold_24329	2E-31	95.5	111	8.00E-44
BC18-c01	12		BC40q10_c23trans_contig9	scaffold_24329	2E-31	97.3	149	2.00E-66
BC18-c01	12	2	BC40q10_c23trans_contig10	superscaffold_376	2E-88			
BC18-c01	15		BC40q10_c23_contig1	superscaffold_633	0	87.4	198	3.00E-55
BC18-c01	16	2	BC18q10_c01trans_contig5	scaffold_6208	5E-13	96.5	260	5.00E-119
BC18-c01	16	2	BC40q10_c23trans_contig15	scaffold_6208	5E-13	95.6	249	1.00E-109
BC18-c01	16		BC40q10_c23trans_contig19	scaffold_6208	5E-13	95.9	97	1.00E-36
BC18-c01	17		BC18q10_c01_contig3	superscaffold_1033	1E-132	99.3	305	3.00E-156
BC18-c01	19		BC40q10_c23trans_contig12	SFRU_RICE_002423	0	92.7	96	9.00E-32
BC18-c01	21		BC18q10_c01trans_contig3	scaffold_9363	5E-	96.4	110	6.00E-45

					113			
BC18-c01	21		BC18q10_c01trans_contig9	scaffold_9363	5E-113	98.2	169	3.00E-79
BC18-c01	24	2	BC40q10_c23trans_contig17	scaffold_1	0	99.1	107	3.00E-48
BC18-c01	24	2	BC40q10_c23_contig2	scaffold_6298	4E-13			4.00E-47
BC18-c01	25		BC40q10_c23trans_contig16	scaffold_9864	0	100.0	171	3.00E-85
BC18-c01	28	2	BC40q10_c23trans_contig14	scaffold_722	1E-37	95.2	188	1.00E-79
BC18-c01	28		BC40q10_c23trans_contig4	superscaffold_75	5E-91	95.5	199	1.00E-83
BC18-c01	25 & 28	2	BC40q10_c23_contig2	superscaffold_194		97.5	122	3.00E-53
BC18-c01	25 & 28		BC40q10_c23_contig5	superscaffold_194		97.3	185	1.00E-84
BC18-c02	4		BC40q10_c17_contig9	scaffold_15885	2E-30	92.9	56	3.00E-14
BC18-c02	9		BC40q10_c17_contig10	scaffold_8993	0	93.3	89	5.00E-30
BC18-c02	14	2	BC18q10_c03trans_contig2	scaffold_183	1E-174			
BC18-c02	14	2	BC18q10_c03_contig1	scaffold_1892	3E-44			
BC18-c02	14	2	BC18q10_c03trans_contig2	scaffold_280	0	86.8	280	4.00E-81
BC18-c02	14	2	BC18q10_c03_contig1	scaffold_7229	7E-67	99.5	211	9.00E-106
BC18-c02	15		BC18q10_c02_contig11	scaffold_10118	3E-66	91.1	135	2.00E-45
BC18-c02	15		BC18q10_c02_contig12	scaffold_10118	3E-66	92.4	158	4.00E-58
BC18-c02	15		BC18q10_c02_contig9	scaffold_3534	0	95.2	439	0.0
BC18-c02	25		BC18q10_c02_contig1	scaffold_11521	1E-36	94.6	110	1.00E-41
BC18-c02	25		BC40q10_c17_contig3	scaffold_11521	1E-36	91.9	123	4.00E-42
BC18-c02	25		BC40q10_c17_contig5	scaffold_1278	7E-96	92.5	133	5.00E-46
BC18-c02	25		BC40q10_c17trans_contig8	scaffold_1278	7E-96	91.4	104	9.00E-33
BC18-c02	25		BC18q10_c02_contig4	scaffold_13056	1E-34	94.7	170	1.00E-69
BC18-c02	25		BC18q10_c02_contig6	scaffold_13056	1E-34	97.7	129	5.00E-57
BC18-c02	25		BC40q10_c17_contig7	scaffold_34805	8E-25	89.1	110	4.00E-31
BC18-c02	25		BC40q10_c17_contig2	scaffold_4573	0	87.1	140	5.00E-37
BC18-c02	25		BC18q10_c02_contig8	scaffold_5684	6E-78	96.1	155	2.00E-66
BC18-c02	25		BC40q10_c17_contig12	scaffold_5684	6E-78	96.1	155	2.00E-66
BC18-c02	25		BC40q10_c17trans_contig12	scaffold_577	0	94.9	117	2.00E-45
BC18-c02	25		BC40q10_c17_contig1	scaffold_6053	2E-111	97.0	100	4.00E-41
BC18-c02	25		BC40q10_c17_contig8	scaffold_6627	1E-54	99.0	104	1.00E-46
BC18-c02	25		BC18q10_c02_contig10	scaffold_7664	5E-31	89.8	108	6.00E-31
BC18-c02	25		BC18q10_c02trans_contig1	scaffold_7664	5E-31	91.4	280	6.00E-104
BC18-c02	25		BC18q10_c02_contig3	scaffold_8666	5E-33	94.1	152	9.00E-60
BC18-c02	25		BC18q10_c02_contig7	SFRU_RICE_000641	2E-116	100.0	104	2.00E-48
BC18-c02	25		BC40q10_c17trans_contig3	superscaffold_1210	2E-53	97.9	95	5.00E-40
BC18-c02	25		BC40q10_c17trans_contig5	superscaffold_1210	2E-53	96.3	109	2.00E-44
BC18-c02	28		BC40q10_c17_contig13	scaffold_722	1E-37	94.2	120	2.00E-45
BC18-c03	4		BC40q10_c18trans_contig1	superscaffold_468	6E-157	92.9	226	3.00E-86

BC18-c03	14	2	BC40q10_c18_contig2	scaffold_1139	3E-33	99.3	142	1.00E-67
BC18-c03	14	2	BC40q10_c18_contig3	scaffold_1892	3E-44			
BC18-c03	14	2	BC40q10_c18_contig2	scaffold_235	2E-36			1.00E-67
BC18-c03	14	2	BC40q10_c18_contig3	scaffold_7229	7E-67	99.5	211	9.00E-106
BC18-c03	14		BC40q10_c18trans_contig3	scaffold_8388	1E-34	94.3	122	5.00E-46
BC18-c03	18		BC40q10_c18_contig6	superscaffold_1125	1E-76	98.2	113	6.00E-50
BC18-c03	18		BC40q10_c18_contig8	superscaffold_1125	1E-76	98.4	253	5.00E-124
BC18-c03	23		BC40q10_c18_contig5	SFRU_RICE_004426	4E-18	85.7	70	5.00E-11
BC18-c03	24		BC40q10_c18_contig7	scaffold_2271	1E-130	83.6	250	1.00E-55
BC18-c03	25		BC40q10_c18trans_contig2	scaffold_11728	1E-23	86.9	130	2.00E-33
BC18-c04	16		BC40q10_c02trans_contig3	scaffold_1450	1E-107	98.5	196	4.00E-94
BC18-c04	16		BC40q10_c02trans_contig4	scaffold_1450	1E-107	96.5	114	1.00E-46
BC18-c04	16		BC40q10_c02_contig4	scaffold_43652	2E-23	94.0	133	6.00E-51
BC18-c04	16		BC40q10_c02_contig3	superscaffold_724	7E-136	92.7	124	2.00E-44
BC18-c04	16		BC18q10_c04trans_contig1	superscaffold_969	2E-160	96.2	132	4.00E-55
BC18-c04	16	2	BC18q10_c04trans_contig6	superscaffold_969	2E-160	97.4	189	6.00E-87
BC18-c04	17	bad	BC40q10_c02trans_contig1	SFRU_RICE_023912	1E-48	79.1	311	4.00E-45
BC18-c04	23		BC18q10_c04trans_contig2	SFRU_RICE_003538	4E-29	86.6	97	4.00E-21
BC18-c05	3	2	BC18q10_c05_contig5	scaffold_9547	3E-79			
BC18-c05	3	2	BC18q10_c05_contig5	superscaffold_1050	0	97.1	104	2.00E-43
BC18-c05	3		BC18q10_c05_contig2	superscaffold_886	0	98.1	107	1.00E-46
BC18-c05	5		BC18q10_c05trans_contig1	superscaffold_84	0	99.3	301	6.00E-154
BC18-c05	6	2	BC40q10_c05_contig16	scaffold_11443	2E-25			
BC18-c05	6	2	BC40q10_c05_contig15	scaffold_3625	7E-16			
BC18-c05	6	2	BC40q10_c05_contig16	scaffold_5988	2E-36	100.0	130	2.00E-62
BC18-c05	6	2	BC40q10_c05_contig15	superscaffold_1093	7E-33	95.2	105	2.00E-40
BC18-c05	8		BC40q10_c05trans_contig9	scaffold_4055	8E-103	98.0	303	2.00E-148
BC18-c05	8		BC40q10_c05_contig13	scaffold_583	2E-59	85.0	180	5.00E-43
BC18-c05	8		BC40q10_c05_contig7	scaffold_583	2E-59	95.3	149	2.00E-60
BC18-c05	8		BC40q10_c05_contig8	scaffold_583	2E-59	89.2	120	1.00E-32
BC18-c05	8		BC40q10_c05_contig4	scaffold_8236	1E-28	100.0	134	7.00E-65
BC18-c05	8		BC40q10_c05trans_contig7	scaffold_8236	1E-28	83.2	279	4.00E-56
BC18-c05	11	vs	BC40q10_c05_contig6	scaffold_2866	1E-20	100.0	44	1.00E-14
BC18-c05	15		BC40q10_c05trans_contig10	superscaffold_1036	0	93.6	204	4.00E-79
BC18-c05	16		BC40q10_c05trans_contig1	superscaffold_354	0	85.5	220	1.00E-54
BC18-c05	16		BC40q10_c05_contig17	superscaffold_831	1E-21	99.6	251	6.00E-128
BC18-c05	20		BC40q10_c05trans_contig3	scaffold_25506	1E-22	89.2	74	1.00E-17
BC18-c05	20		BC40q10_c05trans_contig8	scaffold_25602	1E-22	100.0	28	6.00E-06
BC18-c05	20		BC40q10_c05_contig9	scaffold_36821	2E-11	92.6	108	4.00E-37
BC18-c05	20		BC40q10_c05_contig3	scaffold_6173	7E-63	100.0	119	1.00E-56

BC18-c05	20		BC40q10_c05_contig5	scaffold_6173	7E-63	99.2	122	1.00E-56
BC18-c05	20		BC18q10_c05_contig4	scaffold_6323	4E-40	97.2	108	2.00E-45
BC18-c05	20		BC40q10_c05_contig1	scaffold_7655	3E-108	92.1	139	3.00E-49
BC18-c05	20		BC40q10_c05_contig12	SFRU_RICE_004799	1E-24	100.0	124	2.00E-59
BC18-c05	20		BC40q10_c05_contig10	superscaffold_460	1E-126	99.4	167	2.00E-81
BC18-c05	23		BC40q10_c05trans_contig2	superscaffold_600	1E-120	98.4	250	2.00E-122
BC18-c06	8		BC18q10_c06trans_contig1	scaffold_4055	8E-103	98.3	177	1.00E-83
BC18-c06	8		BC18q10_c06trans_contig4	scaffold_4055	8E-103	97.1	174	1.00E-78
BC18-c06	8		BC18q10_c06_contig1	scaffold_91	1E-51	96.9	64	1.00E-22
BC18-c06	8		BC18q10_c06_contig2	scaffold_91	1E-51	99.1	227	2.00E-112
BC18-c06	18		BC18q10_c06_contig6	superscaffold_823	2E-151	90.2	92	5.00E-26
BC18-c06	22		BC40q10_c06_contig1	scaffold_5666	4E-90	97.5	283	1.00E-135
BC18-c06	22		BC40q10_c06_contig6	scaffold_6239	0	97.4	156	2.00E-70
BC18-c06	22		BC40q10_c06trans_contig1	scaffold_6239	0	95.4	259	2.00E-113
BC18-c06	22		BC40q10_c06_contig5	scaffold_730	9E-33	98.7	236	3.00E-116
BC18-c06	22		BC40q10_c06_contig4	superscaffold_430	3E-79	92.0	149	4.00E-53
BC18-c06	22		BC18q10_c06_contig3	superscaffold_701	1E-21	92.3	182	8.00E-66
BC18-c06	22		BC18q10_c06trans_contig2	superscaffold_701	1E-21	93.9	164	1.00E-63
BC18-c06	22		BC18q10_c06trans_contig3	superscaffold_701	1E-21	97.1	206	4.00E-94
BC18-c08	5		BC40q10_c08_contig10	scaffold_777	0	87.1	101	1.00E-22
BC18-c08	5		BC40q10_c08_contig11	scaffold_777	0	95.0	218	7.00E-92
BC18-c08	14	2	BC40q10_c08trans_contig3	scaffold_1892	3E-44			
BC18-c08	14	2	BC40q10_c08trans_contig3	scaffold_7229	7E-67	98.4	375	0
BC18-c08	17		BC40q10_c08trans_contig5	scaffold_7132	6E-54	96.7	122	1.00E-51
BC18-c08	18	3	BC40q10_c08trans_contig8	superscaffold_1100	3E-39	99.5	190	4.00E-94
BC18-c08	19		BC18q10_c08_contig4	scaffold_12179	7E-143	97.6	125	7.00E-55
BC18-c08	19		BC40q10_c08_contig2	scaffold_158	0	93.0	129	8.00E-47
BC18-c08	19	2	BC18q10_c08_contig7	scaffold_3357	7E-112	99.4	161	5.00E-78
BC18-c08	19		BC18q10_c08_contig8	scaffold_3357	7E-112	91.7	133	2.00E-44
BC18-c08	19	2	BC40q10_c08_contig7	scaffold_3357	7E-112	99.4	161	5.00E-78
BC18-c08	19		BC18q10_c08_contig2	scaffold_7592	1E-60	97.4	234	1.00E-109
BC18-c08	19		BC18q10_c08_contig6	SFRU_RICE_004475	3E-31	91.4	105	6.00E-34
BC18-c08	19		BC18q10_c08_contig3	SFRU_RICE_007174	7E-67	88.8	98	1.00E-26
BC18-c08	19		BC40q10_c08_contig8	superscaffold_107	2E-109	97.0	232	2.00E-107
BC18-c08	19		BC40q10_c08_contig9	superscaffold_113	0	97.3	218	3.00E-101
BC18-c08	19	2	BC40q10_c08_contig7	superscaffold_668	1E-76			
BC18-c08	19		BC18q10_c08trans_contig1	superscaffold_896	1E-110	93.3	75	1.00E-23
BC18-c08	19		BC18q10_c08trans_contig2	superscaffold_896	1E-110	96.2	130	3.00E-54

BC18-c08	22	2	BC18q10_c08_contig7	superscaffold_66	0			
BC18-c08	23	3	BC40q10_c08trans_contig8	scaffold_410	4E-175			1.00E-83
BC18-c08	24		BC40q10_c08trans_contig7	scaffold_1	0	88.9	189	6.00E-57
BC18-c08	27	2	BC40q10_c08trans_contig1	scaffold_13102	2E-24			
BC18-c08	27	2	BC40q10_c08trans_contig1	superscaffold_1097	8E-79	91.0	200	4.00E-70
BC18-c08	28	3	BC40q10_c08trans_contig8	superscaffold_1103	5E-37			2.00E-92
BC18-c09	5		BC40q10_c09_contig5	scaffold_1270	1E-130	98.7	159	8.00E-75
BC18-c09	5		BC40q10_c09trans_contig17	scaffold_13111	4E-40	95.8	71	5.00E-25
BC18-c09	5		BC40q10_c09trans_contig1	scaffold_14779	5E-81	100.0	121	1.00E-57
BC18-c09	5		BC18q10_c09trans_contig1	scaffold_24882	1E-64	99.5	212	2.00E-106
BC18-c09	5		BC40q10_c09_contig2	scaffold_2538	3E-87	99.1	232	1.00E-115
BC18-c09	5		BC40q10_c09trans_contig6	scaffold_3565	1E-34	100.0	109	4.00E-51
BC18-c09	5		BC40q10_c09trans_contig5	scaffold_4752	9E-43	97.4	114	8.00E-49
BC18-c09	5		BC40q10_c09trans_contig7	scaffold_4752	9E-43	100.0	112	1.00E-52
BC18-c09	5		BC40q10_c09trans_contig9	scaffold_4752	9E-43	94.5	73	9.00E-24
BC18-c09	5		BC40q10_c09trans_contig3	scaffold_8188	3E-84	93.9	132	9.00E-49
BC18-c09	5		BC40q10_c09trans_contig19	scaffold_9763	5E-26	97.3	110	4.00E-46
BC18-c09	5		BC18q10_c09trans_contig3	SFRU_RICE_005226	2E-92	95.9	121	2.00E-49
BC18-c09	5		BC40q10_c09trans_contig10	superscaffold_1147	1E-47	92.6	122	3.00E-43
BC18-c09	5		BC18q10_c09trans_contig2	superscaffold_133	2E-120	95.3	148	3.00E-59
BC18-c09	5		BC40q10_c09trans_contig15	superscaffold_133	2E-120	95.3	148	3.00E-59
BC18-c09	5		BC40q10_c09_contig3	superscaffold_84	0	99.3	301	6.00E-154
BC18-c09	5		BC40q10_c09trans_contig12	superscaffold_913	5E-153	98.3	114	2.00E-50
BC18-c09	7		BC18q10_c09_contig5	scaffold_230	1E-35	100.0	67	2.00E-27
BC18-c09	10		BC40q10_c09_contig4	superscaffold_870	7E-116	82.5	171	1.00E-30
BC18-c09	16		BC18q10_c09_contig3	scaffold_6208	5E-13	94.3	262	4.00E-110
BC18-c09	22		BC40q10_c09trans_contig13	scaffold_8948	4E-141	94.4	214	4.00E-89
BC18-c09	26		BC18q10_c09_contig4	scaffold_468	1E-21	98.2	168	4.00E-78
BC18-c09	27		BC40q10_c09trans_contig8	scaffold_586	0	98.2	110	3.00E-48
BC18-c10	1		BC40q10_c11trans_contig1	scaffold_7564	9E-67	100.0	143	8.00E-70
BC18-c10	1		BC40q10_c11trans_contig4	scaffold_7564	9E-67	97.5	118	5.00E-51
BC18-c10	3		BC40q10_c11trans_contig3	superscaffold_886	0	99.4	331	1.00E-170
BC18-c10	5	2	BC40q10_c11_contig13	scaffold_185	0			6.00E-56
BC18-c10	7		BC40q10_c11_contig7	scaffold_27649	2E-45	95.7	161	4.00E-68
BC18-c10	8		BC40q10_c11_contig14	SFRU_RICE_002566	2E-39	87.7	138	5.00E-36
BC18-c10	24	2	BC40q10_c11_contig13	scaffold_2087	2E-144	100.0	146	2.00E-71
BC18-c10	28		BC40q10_c11_contig16	scaffold_17507	3E-27	97.3	149	2.00E-66
BC18-c10	28		BC40q10_c11_contig5	scaffold_37137	4E-30	95.4	129	5.00E-52
BC18-c10	28	2	BC40q10_c11_contig4	scaffold_39475	1E-41	98.1	106	5.00E-46
BC18-c10	28		BC40q10_c11_contig3	superscaffold_1189	0	99.1	319	3.00E-162

BC18-c10	28		BC18q10_c10_contig1	superscaffold_75	5E-91	95.9	145	8.00E-60
BC18-c10	28		BC40q10_c11_contig1	superscaffold_75	5E-91	95.0	160	7.00E-66
BC18-c10	28		BC40q10_c11_contig8	superscaffold_75	5E-91	97.0	101	1.00E-41
BC18-c10	11 &18		BC40q10_c11_contig11	scaffold_598		91.9	99	3.00E-32
BC18-c11	25 & 28		BC18q10_c11trans_contig1	superscaffold_194		99.4	328	6.00E-169
BC18-c12	23		BC18q10_c12_contig1	scaffold_462	0	88.0	83	9.00E-18
BC18-c12	23		BC40q10_c13_contig1	scaffold_462	0	83.6	146	2.00E-30
BC18-c13	7		BC18q10_c13_contig6	scaffold_8962	4E-38	88.9	117	4.00E-32
BC18-c13	18	3	BC40q10_c14_contig6	superscaffold_1100	3E-39			2.00E-71
BC18-c13	18		BC18q10_c13_contig5	superscaffold_239	0	96.5	345	4.00E-161
BC18-c13	18		BC18q10_c13_contig7	superscaffold_515	0	95.7	115	2.00E-45
BC18-c13	18		BC40q10_c14_contig1	superscaffold_618	2E-46	98.0	153	2.00E-70
BC18-c13	23	3	BC40q10_c14_contig6	scaffold_410	4E-175			2.00E-71
BC18-c13	28	3	BC40q10_c14_contig6	superscaffold_1103	5E-37	99.3	152	4.00E-73
BC18-c14	7		BC40q10_c16trans_contig6	superscaffold_1108	3E-26	89.0	209	3.00E-67
BC18-c14	9		BC18q10_c14_contig6	superscaffold_760	2E-52	90.5	42	9.00E-07
BC18-c14	10		BC18q10_c14_contig11	scaffold_1144	4E-140	99.1	115	1.00E-52
BC18-c14	10		BC18q10_c14_contig13	scaffold_1144	4E-140	100.0	105	7.00E-49
BC18-c14	10		BC40q10_c16trans_contig2	scaffold_1194	3E-67	100.0	124	2.00E-59
BC18-c14	10		BC18q10_c14_contig10	scaffold_2074	1E-54	98.6	217	1.00E-104
BC18-c14	10		BC40q10_c16_contig4	scaffold_228	0	97.4	194	1.00E-89
BC18-c14	10		BC18q10_c14_contig18	scaffold_2775	4E-70	95.1	329	5.00E-145
BC18-c14	10		BC40q10_c16_contig5	scaffold_2775	4E-70	95.1	329	5.00E-145
BC18-c14	10		BC18q10_c14_contig17	scaffold_2884	1E-11	98.2	332	6.00E-164
BC18-c14	10		BC18q10_c14_contig5	scaffold_5534	0	100.0	120	4.00E-57
BC18-c14	10		BC18q10_c14trans_contig2	scaffold_5534	0	94.7	150	8.00E-60
BC18-c14	10		BC18q10_c14trans_contig3	SFRU_RICE_008443	0	89.7	107	1.00E-31
BC18-c14	10		BC40q10_c16trans_contig1	superscaffold_486	0	98.3	115	5.00E-51
BC18-c14	10		BC40q10_c16trans_contig3	superscaffold_486	0	99.1	107	3.00E-48
BC18-c14	15		BC18q10_c14_contig2	superscaffold_462	0	97.0	99	1.00E-40
BC18-c14	24		BC18q10_c14_contig1	scaffold_21147	1E-13	87.5	72	6.00E-15
BC18-c14	23 &27		BC40q10_c16trans_contig7	superscaffold_1011		98.0	99	3.00E-42
BC18-c15	7		BC40q10_c01trans_contig5	scaffold_8962	4E-38	96.2	131	3.00E-54
BC18-c15	7		BC40q10_c01trans_contig2	SFRU_RICE_008146	3E-28	100.0	166	1.00E-82
BC18-c15	8		BC40q10_c01_contig4	scaffold_14728	1E-132	83.7	202	6.00E-43
BC18-c15	8		BC18q10_c15_contig1	scaffold_65	0	85.4	123	5.00E-28

BC18-c15	8		BC40q10_c01_contig11	scaffold_91	1E-51	99.1	227	2.00E-112
BC18-c15	8		BC40q10_c01_contig8	scaffold_91	1E-51	96.9	64	1.00E-22
BC18-c15	8		BC40q10_c01_contig12	superscaffold_986	2E-153	93.1	216	2.00E-83
BC18-c15	8		BC40q10_c01_contig19	superscaffold_986	2E-153	95.2	209	5.00E-88
BC18-c15	14	vs	BC40q10_c01_contig10	scaffold_1892	3E-44	100.0	39	4.00E-12
BC18-c15	14		BC40q10_c01_contig13	scaffold_1892	3E-44	97.0	165	1.00E-73
BC18-c15	14		BC40q10_c01_contig17	scaffold_1892	3E-44	93.7	95	3.00E-33
BC18-c15	14		BC40q10_c01trans_contig4	scaffold_1892	3E-44	98.1	310	3.00E-152
BC18-c15	17		BC40q10_c01trans_contig1	scaffold_42011	7E-24	96.2	239	1.00E-105
BC18-c15	22		BC40q10_c01trans_contig3	superscaffold_430	3E-79	92.9	141	5.00E-52
BC18-c15	24		BC40q10_c01_contig16	scaffold_1630	2E-68	93.3	224	8.00E-87
BC18-c15	27	2	BC18q10_c15_contig2	SFRU_RICE_008260	2E-147			
BC18-c15	27	2	BC18q10_c15_contig2	SFRU_RICE_020820	3E-28	88.7	115	5.00E-32
BC18-c16	9		BC40q10_c03_contig4	scaffold_8938	6E-50	87.3	110	6.00E-26
BC18-c16	9		BC40q10_c03_contig9	scaffold_8938	6E-50	99.3	144	1.00E-68
BC18-c16	9		BC18q10_c16trans_contig2	SFRU_RICE_016343	0	90.3	154	2.00E-50
BC18-c16	9		BC18q10_c16trans_contig1	superscaffold_1206	0	97.6	169	2.00E-77
BC18-c16	9		BC40q10_c03_contig2	superscaffold_760	2E-52	94.5	127	2.00E-49
BC18-c16	9		BC40q10_c03_contig7	superscaffold_94	0	98.1	104	5.00E-45
BC18-c16	22	2	BC40q10_c03trans_contig1	scaffold_9330	1E-40			
BC18-c16	22	2	BC40q10_c03trans_contig1	superscaffold_37	1E-41	90.9	164	6.00E-55
BC18-c16	22		BC40q10_c03trans_contig2	superscaffold_701	1E-21	93.9	164	1.00E-63
BC18-c16	22		BC40q10_c03trans_contig3	superscaffold_701	1E-21	97.1	206	4.00E-94
BC18-c16	24	2	BC40q10_c03_contig10	scaffold_6298	4E-13			4.00E-23
BC18-c16	25 &28	2	BC40q10_c03_contig10	superscaffold_194		98.5	136	1.00E-62
BC18-c17	1		BC18q10_c17_contig12	scaffold_5532	9E-110	97.0	131	2.00E-56
BC18-c17	1		BC18q10_c17_contig27	scaffold_5532	9E-110	96.8	94	8.00E-38
BC18-c17	4		BC18q10_c17_contig22	scaffold_105	3E-133	89.3	150	8.00E-45
BC18-c17	4		BC18q10_c17_contig24	scaffold_105	3E-133	91.2	260	2.00E-92
BC18-c17	4	2	BC40q10_c20trans_contig3	scaffold_5327	3E-21	96.1	51	4.00E-15
BC18-c17	6		BC18q10_c17trans_contig1	scaffold_20856	2E-44	94.0	199	1.00E-78
BC18-c17	6		BC18q10_c17trans_contig4	scaffold_20856	2E-44	97.0	131	5.00E-56
BC18-c17	7		BC18q10_c17_contig14	superscaffold_336	0	100.0	144	2.00E-70
BC18-c17	9		BC40q10_c20_contig11	scaffold_16039	2E-119	99.0	103	4.00E-46
BC18-c17	15		BC18q10_c17_contig18	scaffold_5685	1E-33	100.0	221	5.00E-113
BC18-c17	15		BC40q10_c20_contig1	scaffold_5685	1E-33	100.0	169	3.00E-84
BC18-c17	15		BC40q10_c20_contig13	scaffold_5685	1E-33	99.0	103	4.00E-46
BC18-c17	15		BC18q10_c17_contig9	superscaffold_390	5E-101	90.9	187	5.00E-63

BC18-c17	15		BC18q10_c17_contig25	superscaffold_493	2E-175	95.0	99	5.00E-37
BC18-c17	16	2	BC18q10_c17trans_contig11	scaffold_6208	5E-13	96.4	195	2.00E-86
BC18-c17	16		BC18q10_c17trans_contig12	scaffold_6208	5E-13	95.7	116	1.00E-46
BC18-c17	16		BC40q10_c20_contig9	superscaffold_202	0	92.5	106	2.00E-34
BC18-c17	16		BC40q10_c20_contig16	superscaffold_354	0	94.1	101	1.00E-36
BC18-c17	17		BC18q10_c17_contig5	scaffold_19569	0	100.0	128	1.00E-61
BC18-c17	17		BC40q10_c20_contig18	scaffold_34618	2E-16	97.0	101	1.00E-41
BC18-c17	17	2	BC40q10_c20trans_contig2	scaffold_40254	5E-26			
BC18-c17	17		BC18q10_c17_contig17	scaffold_42011	7E-24	93.4	152	5.00E-57
BC18-c17	17		BC18q10_c17_contig8	scaffold_42011	7E-24	93.2	118	4.00E-42
BC18-c17	17		BC40q10_c20trans_contig1	scaffold_42011	7E-24	89.3	234	1.00E-75
BC18-c17	17	2	BC40q10_c20trans_contig2	scaffold_42011	7E-24	95.4	87	7.00E-32
BC18-c17	17		BC40q10_c20_contig10	scaffold_7124	3E-15	99.0	99	6.00E-44
BC18-c17	17		BC18q10_c17trans_contig7	scaffold_7132	6E-54	100.0	125	7.00E-60
BC18-c17	17		BC18q10_c17trans_contig9	scaffold_7132	6E-54	99.2	397	0.0
BC18-c17	17		BC40q10_c20_contig4	scaffold_7665	3E-65	99.1	112	4.00E-51
BC18-c17	17		BC18q10_c17_contig4	scaffold_8364	8E-17	98.1	107	1.00E-46
BC18-c17	17	vs	BC40q10_c20_contig17	scaffold_8364	8E-17	98.0	49	5.00E-16
BC18-c17	17		BC40q10_c20_contig12	SFRU_RICE_010738	2E-38	99.5	187	1.00E-92
BC18-c17	17		BC18q10_c17_contig2	superscaffold_725	0	100.0	134	7.00E-65
BC18-c17	17		BC40q10_c20_contig8	superscaffold_725	0	98.2	113	2.00E-49
BC18-c17	18		BC18q10_c17_contig21	scaffold_25799	3E-40	97.4	153	1.00E-68
BC18-c17	22	2	BC40q10_c20trans_contig3	superscaffold_495	2E-14			2.00E-08
BC18-c17	23		BC18q10_c17trans_contig5	superscaffold_604	1E-46	97.9	193	8.00E-91
BC18-c17	23		BC18q10_c17trans_contig6	superscaffold_604	1E-46	93.2	147	1.00E-53
BC18-c17	28		BC18q10_c17_contig23	scaffold_11686	4E-44	99.1	112	4.00E-51
BC18-c17	28		BC18q10_c17_contig26	scaffold_11686	4E-44	99.1	219	2.00E-108
BC18-c18	9		BC40q10_c30trans_contig2	scaffold_188	0	89.4	132	7.00E-40
BC18-c18	11		BC40q10_c30trans_contig1	scaffold_5087	3E-59	93.8	336	7.00E-139
BC18-c18	24		BC18q10_c18trans_contig2	scaffold_1685	1E-24	85.0	240	5.00E-59
BC18-c19	7	2	BC18q10_c19_contig5	scaffold_1312	0	98.0	201	1.00E-94
BC18-c19	7	2	BC18q10_c19_contig6	scaffold_1312	0			
BC18-c19	7		BC40q10_c25_contig11	scaffold_230	1E-35	93.9	228	2.00E-92
BC18-c19	7		BC40q10_c25_contig1	scaffold_3280	3E-20	100.0	251	1.00E-129
BC18-c19	7		BC40q10_c25_contig6	scaffold_344	2E-131	90.3	113	6.00E-35
BC18-c19	7	2	BC18q10_c19_contig5	superscaffold_782	0			
BC18-c19	7	2	BC18q10_c19_contig6	superscaffold_782	0	100.0	89	4.00E-40
BC18-c19	7		BC18q10_c19_contig2	superscaffold_800	6E-176	96.7	92	1.00E-36
BC18-c20	3		BC40q10_c21_contig5	SFRU_RICE_028224	2E-15	96.3	80	5.00E-30
BC18-c20	4		BC18q10_c20_contig11	superscaffold_161	0	95.3	191	9.00E-81
BC18-c20	6	2	BC40q10_c21_contig13	scaffold_521	2E-33	99.6	267	8.00E-137

BC18-c20	6	2	BC40q10_c21_contig16	scaffold_521	2E-33	97.4	117	2.00E-50
BC18-c20	10	2	BC40q10_c21_contig13	scaffold_16	0			8.00E-137
BC18-c20	10	2	BC40q10_c21_contig16	scaffold_16	0			2.00E-50
BC18-c20	12		BC18q10_c20_contig2	scaffold_12330	3E-73	93.2	103	4.00E-36
BC18-c20	12		BC18q10_c20_contig6	scaffold_12330	3E-73	88.2	237	1.00E-70
BC18-c20	12		BC40q10_c21_contig1	scaffold_12330	3E-73	93.2	103	4.00E-36
BC18-c20	12		BC40q10_c21_contig3	scaffold_12330	3E-73	88.2	237	1.00E-70
BC18-c20	12	2	BC40q10_c21_contig14	scaffold_1537	3E-46	99.3	139	6.00E-66
BC18-c20	12		BC18q10_c20_contig1	scaffold_1558	0	99.3	151	1.00E-72
BC18-c20	12	2	BC40q10_c21_contig14	scaffold_1595	1E-46			
BC18-c20	12	2	BC40q10_c21_contig10	scaffold_33339	5E-43	87.6	129	1.00E-32
BC18-c20	12		BC40q10_c21_contig2	scaffold_33339	5E-43	98.0	100	9.00E-43
BC18-c20	12	2	BC40q10_c21_contig10	scaffold_33671	4E-71			
BC18-c20	12		BC18q10_c20_contig7	scaffold_4990	2E-26	97.5	121	4.00E-52
BC18-c20	12		BC18q10_c20trans_contig1	SFRU_RICE_003701	2E-66	99.0	190	5.00E-92
BC18-c20	12		BC40q10_c21_contig4	superscaffold_1158	0	99.4	155	8.00E-75
BC18-c20	12		BC40q10_c21_contig6	superscaffold_1158	0	100.0	132	9.00E-64
BC18-c20	15		BC40q10_c21trans_contig2	superscaffold_42	5E-101	99.3	133	1.00E-62
BC18-c20	17		BC18q10_c20trans_contig6	superscaffold_571	0	93.9	197	4.00E-79
BC18-c20	17		BC18q10_c20trans_contig7	superscaffold_571	0	90.8	120	8.00E-39
BC18-c20	19		BC18q10_c20trans_contig2	scaffold_7245	0	99.2	239	1.00E-119
BC18-c20	22		BC40q10_c21_contig15	superscaffold_304	2E-47	99.1	213	3.00E-105
BC18-c21	11	2	BC40q10_c22trans_contig6	scaffold_1285	1E-13			2.00E-67
BC18-c21	11	2	BC40q10_c22trans_contig6	scaffold_2688	0	91.9	234	2.00E-87
BC18-c21	11		BC18q10_c21trans_contig2	scaffold_7463	5E-25	89.2	158	6.00E-47
BC18-c21	11		BC40q10_c22trans_contig4	SFRU_RICE_004909	4E-39	91.7	72	9.00E-20
BC18-c21	11		BC40q10_c22_contig1	SFRU_RICE_005554	7E-33	93.3	104	1.00E-36
BC18-c21	11		BC40q10_c22_contig3	SFRU_RICE_012770	1E-34	100.0	104	2.00E-48
BC18-c21	11		BC18q10_c21trans_contig4	SFRU_RICE_012847	1E-140	93.2	74	2.00E-23
BC18-c21	23	2	BC18q10_c21_contig4	scaffold_1857	2E-27	93.6	62	1.00E-17
BC18-c22	9		BC18q10_c22trans_contig6	SFRU_RICE_016343	0	92.9	113	5.00E-40
BC18-c22	15		BC18q10_c22trans_contig13	scaffold_9543	1E-91	97.3	146	8.00E-65
BC18-c22	16	2	BC18q10_c22trans_contig10	scaffold_6208	5E-13	98.3	120	3.00E-53
BC18-c22	22		BC18q10_c22trans_contig1	scaffold_266	9E-135	94.4	108	2.00E-40
BC18-c22	23		BC18q10_c22trans_contig2	superscaffold_1052	8E-138	93.6	94	1.00E-31
BC18-c22	24		BC18q10_c22trans_contig8	scaffold_17097	1E-24	98.5	133	6.00E-61
BC18-c22	26		BC18q10_c22trans_contig11	SFRU_RICE_005307	5E-87	100.0	114	6.00E-54
BC18-c23	15		BC40q10_c07trans_contig3	scaffold_1891	0	95.1	102	2.00E-38
BC18-c23	15		BC18q10_c23_contig3	scaffold_21	0	94.8	96	1.00E-34
BC18-c23	15	2	BC18q10_c23trans_contig4	scaffold_242	2E-27			1.00E-41

BC18-c23	15		BC40q10_c07_contig5	scaffold_3534	0	89.6	212	2.00E-67
BC18-c23	15		BC18q10_c23_contig2	scaffold_37847	2E-12	90.3	72	7.00E-19
BC18-c23	15		BC40q10_c07_contig3	scaffold_4848	5E-61	95.0	80	7.00E-28
BC18-c23	15		BC40q10_c07_contig4	scaffold_602	2E-98	96.4	253	1.00E-115
BC18-c23	15		BC40q10_c07trans_contig5	scaffold_9543	1E-91	97.9	192	3.00E-90
BC18-c23	15		BC18q10_c23_contig5	scaffold_9604	4E-58	97.2	177	3.00E-80
BC18-c23	15		BC40q10_c07_contig8	SFRU_RICE_002743	5E-28	91.2	159	3.00E-54
BC18-c23	15		BC40q10_c07trans_contig4	SFRU_RICE_004231	5E-60	100.0	190	7.00E-96
BC18-c23	15		BC40q10_c07trans_contig1	SFRU_RICE_012905	4E-39	100.0	104	2.00E-48
BC18-c23	15		BC40q10_c07_contig10	superscaffold_1157	5E-28	93.2	118	5.00E-42
BC18-c23	15		BC40q10_c07_contig9	superscaffold_493	2E-175	95.5	110	9.00E-43
BC18-c23	15		BC40q10_c07trans_contig2	superscaffold_493	2E-175	94.9	97	7.00E-36
BC18-c23	15		BC40q10_c07_contig1	superscaffold_624	5E-160	87.1	209	1.00E-56
BC18-c23	15		BC40q10_c07_contig7	superscaffold_624	5E-160	93.5	339	5.00E-140
BC18-c23	20	2	BC18q10_c23trans_contig3	scaffold_6397	4E-40			
BC18-c23	25		BC18q10_c23trans_contig1	scaffold_577	0	91.7	277	3.00E-101
BC18-c23	28		BC18q10_c23_contig4	scaffold_722	1E-37	97.9	188	7.00E-88
BC18-c24	7	2	BC40q10_c24trans_contig4	scaffold_230	1E-35			2.00E-25
BC18-c24	11		BC40q10_c24trans_contig3	scaffold_15528	2E-161	91.7	192	1.00E-69
BC18-c24	13		BC18q10_c24_contig1	superscaffold_637	0	97.6	165	9.00E-75
BC18-c25	2	2	BC18q10_c25trans_contig5	SFRU_RICE_010151	3E-26			5.00E-26
BC18-c25	4		BC40q10_c27_contig9	scaffold_2136	1E-146	99.4	171	1.00E-83
BC18-c25	18		BC18q10_c25_contig1	scaffold_3044	5E-84	97.9	96	2.00E-40
BC18-c25	18		BC18q10_c25_contig4	scaffold_3044	5E-84	80.5	164	9.00E-25
BC18-c25	24		BC40q10_c27_contig8	SFRU_RICE_011931	1E-53	95.2	124	7.00E-49
BC18-c25	27	2	BC18q10_c25trans_contig3	scaffold_13102	2E-24			
BC18-c25	27		BC40q10_c27_contig5	scaffold_181	2E-50	95.0	100	3.00E-37
BC18-c25	27		BC18q10_c25trans_contig7	scaffold_52	9E-160	94.8	77	3.00E-26
BC18-c25	27		BC40q10_c27_contig7	scaffold_52	9E-160	100.0	82	4.00E-36
BC18-c25	27		BC18q10_c25trans_contig6	scaffold_607	3E-154	87.5	255	5.00E-74
BC18-c25	27	2	BC18q10_c25trans_contig5	SFRU_RICE_020820	3E-28	100.0	138	4.00E-67
BC18-c25	27	2	BC18q10_c25trans_contig3	superscaffold_1097	8E-79	91.0	199	1.00E-69
BC18-c26	3		BC18q10_c26_X_contig6	superscaffold_111	0	98.8	246	2.00E-121
BC18-c26	6	2	BC18q10_c26_X_contig5	scaffold_521	2E-33	98.6	573	0.0
BC18-c26	10	2	BC18q10_c26_X_contig5	scaffold_16	0			0.00E+00
BC18-c26	11		BC18q10_c26_X_contig18	scaffold_22580	3E-180	98.4	370	0.0
BC18-c26	11		BC40q10_c12trans_contig2	scaffold_395	2E-24	93.0	71	4.00E-21
BC18-c26	17		BC18q10_c26_X_contig8	scaffold_4502	3E-74	97.0	131	3.00E-56

BC18-c26	19		BC18q10_c26_X_contig14	scaffold_158	0	93.0	100	7.00E-34
BC18-c26	19		BC18q10_c26_X_contig15	scaffold_158	0	94.9	138	2.00E-55
BC18-c26	21		BC40q10_c12_contig11	scaffold_13640	2E-18	93.7	126	6.00E-46
BC18-c26	21		BC40q10_c12_contig9	scaffold_13640	2E-18	97.1	208	3.00E-95
BC18-c26	21		BC40q10_c12_contig1	scaffold_3849	1E-10	96.2	132	1.00E-54
BC18-c26	21		BC40q10_c12_contig3	scaffold_7630	3E-25	100.0	113	3.00E-53
BC18-c26	21		BC40q10_c12_contig5	scaffold_9801	8E-148	91.2	204	7.00E-73
BC18-c26	21		BC40q10_c12_contig6	scaffold_9801	8E-148	95.6	183	1.00E-78
BC18-c26	21		BC40q10_c12trans_contig1	SFRU_RICE_004516	0	100.0	104	2.00E-48
BC18-c26	21		BC40q10_c12_contig10	SFRU_RICE_024839	3E-16	94.3	140	7.00E-55
BC18-c28	2	2	BC18q10_c28_contig6	scaffold_2482	2E-34	97.7	128	1.00E-56
BC18-c28	2		BC40q10_c29_contig9	scaffold_3005	5E-13	97.3	224	1.00E-104
BC18-c28	2	2	BC18q10_c28_contig6	scaffold_464	4E-95			
BC18-c28	2		BC18q10_c28_contig1	SFRU_RICE_016681	1E-17	88.5	182	1.00E-53
BC18-c28	7		BC40q10_c29_contig10	scaffold_230	1E-35	100.0	64	1.00E-25
BC18-c28	16	2	BC40q10_c29_contig1	scaffold_6208	5E-13	96.0	151	1.00E-63
BC18-c29	3		BC40q10_c28_contig4	scaffold_234	0	100.0	108	2.00E-50
BC18-c29	10		BC40q10_c28trans_contig1	scaffold_1144	4E-140	100.0	154	7.00E-76
BC18-c29	11		BC18q10_c29_contig11	scaffold_7463	5E-25	95.2	105	2.00E-39
BC18-c29	11		BC40q10_c28_contig7	superscaffold_1110	4E-53	82.4	216	8.00E-43
BC18-c29	19	2	BC18q10_c29trans_contig4	scaffold_445	3E-130	97.6	247	2.00E-117
BC18-c29	19	2	BC18q10_c29trans_contig4	scaffold_519	1E-128			
BC18-c29	23	2	BC18q10_c29_contig1	scaffold_2332	0			
BC18-c29	23	2	BC18q10_c29_contig7	scaffold_2332	0			
BC18-c29	23	2	BC40q10_c28_contig2	scaffold_24206	4E-41	92.9	212	2.00E-82
BC18-c29	23		BC18q10_c29_contig2	scaffold_26848	2E-40	89.6	134	2.00E-41
BC18-c29	23	2	BC40q10_c28_contig2	scaffold_26848	2E-40			
BC18-c29	23		BC18q10_c29_contig10	scaffold_463	4E-66	94.0	117	8.00E-44
BC18-c29	23		BC18q10_c29_contig5	SFRU_RICE_003952	0	91.8	122	2.00E-39
BC18-c29	23		BC40q10_c28_contig6	SFRU_RICE_021239	0	88.5	165	1.00E-48
BC18-c29	23		BC18q10_c29_contig3	superscaffold_1052	8E-138	95.2	124	2.00E-48
BC18-c29	23	2	BC18q10_c29_contig1	superscaffold_1165	0	94.9	98	1.00E-36
BC18-c29	23	2	BC18q10_c29_contig7	superscaffold_1165	0	91.6	142	1.00E-47
BC18-c29	23		BC18q10_c29trans_contig1	superscaffold_511	0	97.1	136	3.00E-59
BC18-c29	23		BC18q10_c29_contig6	superscaffold_755	0	95.9	98	2.00E-38
BC18-c29	28		BC40q10_c28trans_contig2	scaffold_722	1E-37	95.1	142	1.00E-57
BC18-c29	28		BC18q10_c29trans_contig3	superscaffold_75	5E-91	88.7	416	3.00E-133
BC18-c30	6		BC40q10_c10_contig3	scaffold_20747	8E-29	92.9	127	6.00E-46
BC18-c30	6		BC40q10_c10_contig1	scaffold_20856	2E-44	96.6	149	8.00E-65
BC18-c30	6	3	BC40q10_c10trans_contig9	scaffold_5159	3E-31			

BC18-c30	6		BC40q10_c10trans_contig7	scaffold_600	0	88.6	176	5.00E-53
BC18-c30	6		BC40q10_c10trans_contig2	scaffold_828	0	90.1	111	3.00E-33
BC18-c30	6	3	BC40q10_c10trans_contig9	scaffold_828	0	97.8	134	7.00E-60
BC18-c30	6		BC40q10_c10_contig5	superscaffold_102	6E-35	89.3	149	7.00E-46
BC18-c30	6	3	BC40q10_c10trans_contig9	superscaffold_1093	7E-33			
BC18-c30	6		BC40q10_c10trans_contig1	superscaffold_475	0	97.2	249	3.00E-116
BC18-c30	6		BC40q10_c10trans_contig3	superscaffold_475	0	99.3	138	2.00E-65
BC18-c30	6		BC40q10_c10trans_contig6	superscaffold_475	0	94.4	107	7.00E-39
BC18-c30	7	2	BC18q10_c30trans_contig1	superscaffold_895	3E-160	87.8	74	2.00E-15
BC18-c30	7	2	BC18q10_c30trans_contig3	superscaffold_895	3E-160	97.0	201	2.00E-91
BC18-c31	4		BC40q10_c15_contig1	scaffold_102	0	93.5	306	1.00E-125
BC18-c31	4		BC40q10_c15trans_contig1	scaffold_105	3E-133	92.5	80	2.00E-24
BC18-c31	4		BC18q10_c31_contig6	scaffold_54	0	98.8	165	1.00E-78
BC18-c31	4	2	BC18q10_c31_contig1	scaffold_6120	4E-15	87.1	233	4.00E-64
BC18-c31	4		BC18q10_c31_contig2	superscaffold_1028	8E-106	97.1	137	7.00E-60
BC18-c31	4		BC18q10_c31_contig8	superscaffold_161	0	99.0	100	2.00E-44
BC18-c31	4		BC40q10_c15_contig2	superscaffold_161	0	98.3	181	3.00E-85
BC18-c31	4		BC40q10_c15_contig4	superscaffold_161	0	97.0	100	4.00E-41
BC18-c31	4		BC40q10_c15_contig5	superscaffold_161	0	96.3	162	2.00E-70
BC18-c31	4		BC40q10_c15_contig9	superscaffold_161	0	97.1	102	3.00E-42
BC18-c31	4	2	BC18q10_c31_contig1	superscaffold_51	0			
BC18-c31	4		BC18q10_c31_contig12	superscaffold_72	2E-130	96.5	366	2.00E-169
BC18-c31	4		BC18q10_c31_contig5	superscaffold_72	2E-130	90.7	108	3.00E-33
BC18-c31	4		BC18q10_c31_contig7	superscaffold_72	2E-130	99.2	126	3.00E-58
BC18-c31	4		BC18q10_c31trans_contig3	superscaffold_72	2E-130	99.6	268	2.00E-137
BC18-c31	22		BC18q10_c31_contig3	superscaffold_1091	1E-55	94.4	196	4.00E-79
BC18-c31	24	2	BC18q10_c31trans_contig2	scaffold_15445	2E-111			
BC18-c31	24	2	BC18q10_c31trans_contig4	scaffold_15445	2E-111			
BC18-c31	24	2	BC18q10_c31trans_contig2	superscaffold_839	9E-111	100.0	83	1.00E-36
BC18-c31	24	2	BC18q10_c31trans_contig4	superscaffold_839	9E-111	99.1	113	1.00E-51
BC18-c31	28		BC40q10_c15trans_contig3	scaffold_897	3E-32	95.4	87	2.00E-31
BC18-c32	24	vs	BC18q10_c32trans_contig2	scaffold_11156	2E-18	100.0	41	3.00E-13
BC18-c32	24		BC18q10_c32_contig1	scaffold_2316	1E-27	92.0	236	6.00E-88
