# Supplementary material for: Genetic basis of allochronic differentiation in the fall armyworm
Source: BMC Evol Biol. 2017 Mar 6;17:68. doi: 10.1186/s12862-017-0911-5 (PMC5339952; doi:10.1186/s12862-017-0911-5)
Supplement: Additional file 8: — PCR conditions used in the mentioned experiments. (PDF 47 kb) [file 12862_2017_911_MOESM8_ESM.pdf]

Additional file 8.

PCR conditions used in the mentioned experiments.

Experiment	Candidate Gene	PCR components[1]		PCR program	
QTL 1	Vrille	1 µl<br>11.92 µl<br>2 µl<br>2 µl<br>3 µl<br>0.08 µl	DNA<br>dH_2O<br>10x Taq buffer<br>2 mM dNTPs<br>10 mM primer mix[2]<br>Taq polymerase	2 min<br>45 s<br>35x 45 s<br>60 s<br>10 min	94°C<br>94°C<br>T_a[2]<br>72 °C<br>72 °C
Structure analysis of *Vrille*	Vrille	1 µl<br>11.92 µl<br>2 µl<br>2 µl<br>3 µl<br>0.08 µl	DNA<br>dH_2O<br>10x Taq buffer<br>2 mM dNTPs<br>10 mM primer mix[2]<br>Taq polymerase	2 min<br>45 s<br>35x 45 s<br>90 s<br>10 min	94°C<br>94°C<br>T_a[2]<br>72°C<br>72°C
	Vrille (touchdown PCR for degenerate primers)	1 µl<br>11.92 µl<br>2 µl<br>2 µl<br>3 µl<br>0.08 µl	DNA<br>dH_2O<br>10x Taq buffer<br>2 mM dNTPs<br>10 mM primer mix[2]<br>Taq polymerase	3 min<br>30 s<br>28x 30 s<br>60 s<br>30 s<br>23x 30 s<br>60 s	94°C<br>94°C<br>T_a[2] *<br>(decrease<br>by 0,7)<br>72°C<br>94°C<br>Lowest T_a[2]<br>72°C
Expression analysis	Vrille<br>eIF1α	1 µl<br>10 µl<br>1 µl<br>1 µl<br>12 µl	cDNA<br>dH_2O<br>10 mM primer fw<br>10 mM primer rv<br>SYBR Mix[3]	10 min<br>30 s<br>40x 60 s<br>60 s<br>60 s<br>30s<br>30s	90°C<br>95°C<br>58°C<br>72°C<br>95°C<br>58°C<br>95°C

[1]Taq polymerase, dNTPs, buffer and primers were purchased from Metabion, Martinsried, Germany

[2]Primers and corresponding annealing temperatures (T_a) can be found in Table S6

[3] ABsolute Blue QPCR SYBR Green Mix from Thermo Fisher Scientific, Schwerte, Germany
