# Supplementary material for: Genetic basis of allochronic differentiation in the fall armyworm
Source: BMC Evol Biol. 2017 Mar 6;17:68. doi: 10.1186/s12862-017-0911-5 (PMC5339952; doi:10.1186/s12862-017-0911-5)
Supplement: Additional file 9: — Primer combinations and annealing temperatures (Ta). (PDF 71 kb) [file 12862_2017_911_MOESM9_ESM.pdf]

Additional file 9

Primer combinations and annealing temperatures (T_a) of candidate genes.

Experiment	Candidate Gene	Primer	T_a (°C)
QTL 1	Vrille	PC19 Forward: 5'CGACCCAAATGACTACTCTCCT<br>PC19 Reverse: 5' CGTCAGCTTACTCCTCTTGGTT	58
		PC34 Forward: 5' ACCGGCTCATAATTGATCGTT<br>PC34 Reverse: 5' GTCGGTTGCAAAAACTGAATGTC	58
		PC38 Forward: 5' CGG GGC AAC CGA CAA AAA AAT<br>PC38 Reverse: 5' GCGTTCAATAAAAAGGACGCGGATCA	58
		EBOX1 Forward: 5' GATCCGCGTCCTTTTTATTGAAC<br>EBOX1 Reverse: 5'CGAAAGCATCACTCAACACAATG	58
Structure analysis of *Vrille*	Vrille	PC8 Forward: 5'GTCCGCCGAAACATGGTYGCMG<br>PC8 Reverse: 5' GDACTGAACCGGGDGGTTCG	61 -50
		PC19 Forward: 5'CGACCCAAATGACTACTCTCCT<br>PC19 Reverse: 5' CGTCAGCTTACTCCTCTTGGTT	58
		PC21 Forward: 5'CCCTACCAGGAGAGGCTACC<br>PC21 Forward: 5' TCAGTGCTCGWGCSMGCGSG	58
		PC34 Forward: 5' ACCGGCTCATAATTGATCGTT<br>PC34 Reverse: 5' GTCGGTTGCAAAAACTGAATGTC	58
		PC38 Forward: 5' CGG GGC AAC CGA CAA AAA AAT<br>PC38 Reverse: 5' GCGTTCAATAAAAAGGACGCGGATCA	58
		CDS+5' Forward: 5' TGTCACGTGTTTCAAGCATGGTA<br>CDS+5' Reverse: 5' TGTTCTGGTGCATCATGTTCTTC	58
		EBOXBCD Forward: 5' ATTTCACGTTCTTCGATCAC<br>EBOXBCD Reverse: 5' TAAATGCAAATGCACAGAAC	55
		EBOXDE Forward: 5' CTAATCGCGGTTCTAATGAC<br>EBOXDE Reverse: 5' CATTCGAAACTTAAGGTTGC	55
		EBOXFGH Forward: 5' GCAACCTTAAGTTTCGAATG<br>EBOXFGH Reverse: ACGGTGACGACACTCTAAAT	55

		EBOXI Forward: 5’ GATCCGCGTCCTTTTTATTGAAC<br>EBOXI Reverse: 5’CGAAAGCATCACTCAACACAATG	58
		EBOXJ Forward: 5’ ACCGGCTCATAATTGATCGTT<br>EBOXJ Reverse: 5’GCGGATTTCTTCCGTTACAA	58
		EBOXK Forward: 5’ TGTCACGTGTTTCAAGCATGGTA<br>EBOXK Reverse: 5’GTCGGTTGCAAAAACTGAATGTC	58
		DNAwalk1 TSP1: 5’GCGTCAGCTTACTCCTCTTGGTT<br>DNAwalk1 TSP2: 5’GCTGTGCTTTGAGTACGTGGTTC<br>DNAwalk1 TSP3: 5’GCGCTGTCCAAAGAACTCCTTGC	65<br>65<br>66
		DNAwalk2 TSP1: 5’TGAGCCGGTAATACAGGAAGTGTAA<br>DNAwalk2 TSP2: 5’TGCCTATTGTGGCGACTTAGTTTGAT<br>DNAwalk2 TSP3: 5’GCGTTCAATAAAAAGGACGCGGATCA	64.1<br>64.4<br>66.2
Expression analysis	Vrille	Forward: 5’ CTGTGCTTTGAGTACGTGGTTC<br>Reverse: 5’ GCAAACAGAGGGAGTTCATACC	58
	eIF1α	Forward: 5’AGGAGTTGCGTCGTGGTTAC<br>Reverse: 5’ CTTTGATTTCGGCGAACTTG	58
