# Supplementary material for: Genetic basis of allochronic differentiation in the fall armyworm
Source: BMC Evol Biol. 2017 Mar 6;17:68. doi: 10.1186/s12862-017-0911-5 (PMC5339952; doi:10.1186/s12862-017-0911-5)
Supplement: Additional file 10: — Mean onset time of mating for individuals that are homozygous or heterozygous for the QTC Sf_C25. (PDF 107 kb) [file 12862_2017_911_MOESM10_ESM.pdf]

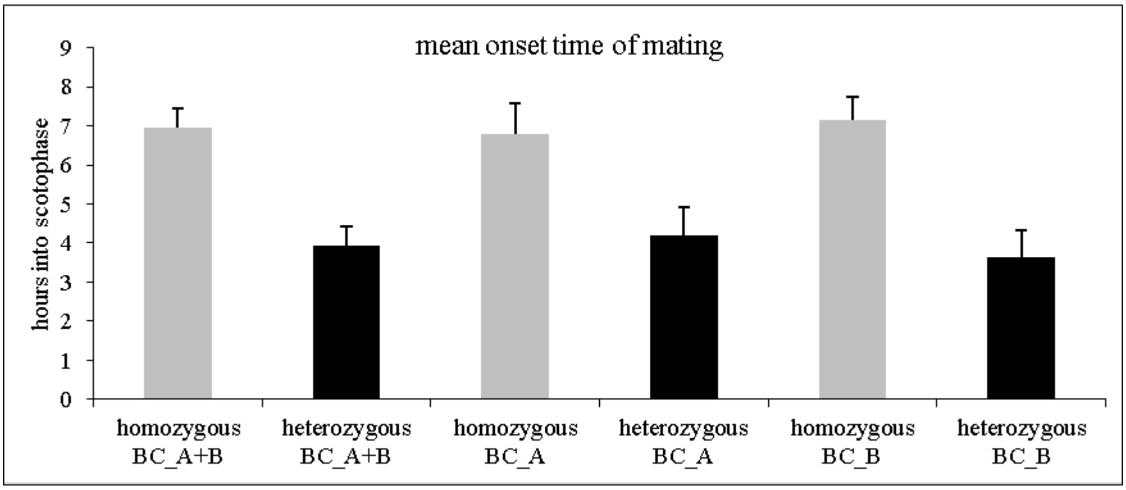

Additional file 10

Mean onset time of mating for individuals that are homozygous (carrying only rice-strain copies) or heterozygous (carrying a corn-strain copy) for the QTL chromosome *Sf*_C25 in both backcrosses analyzed together (left two bars) or analyzed individually (middle two bars BC_A, right two bars BC_B). In all analyses, homozygous individuals with no corn-strain copy mated significantly later than the heterozygous individuals, which is consistent with the strain-specific mating time of rice-strain individuals mating significantly later than corn-strain individuals.
