# Supplementary material for: Genetic basis of allochronic differentiation in the fall armyworm
Source: BMC Evol Biol. 2017 Mar 6;17:68. doi: 10.1186/s12862-017-0911-5 (PMC5339952; doi:10.1186/s12862-017-0911-5)
Supplement: Additional file 11: — LOD scores for all linkage groups. (PDF 36 kb) [file 12862_2017_911_MOESM11_ESM.pdf]

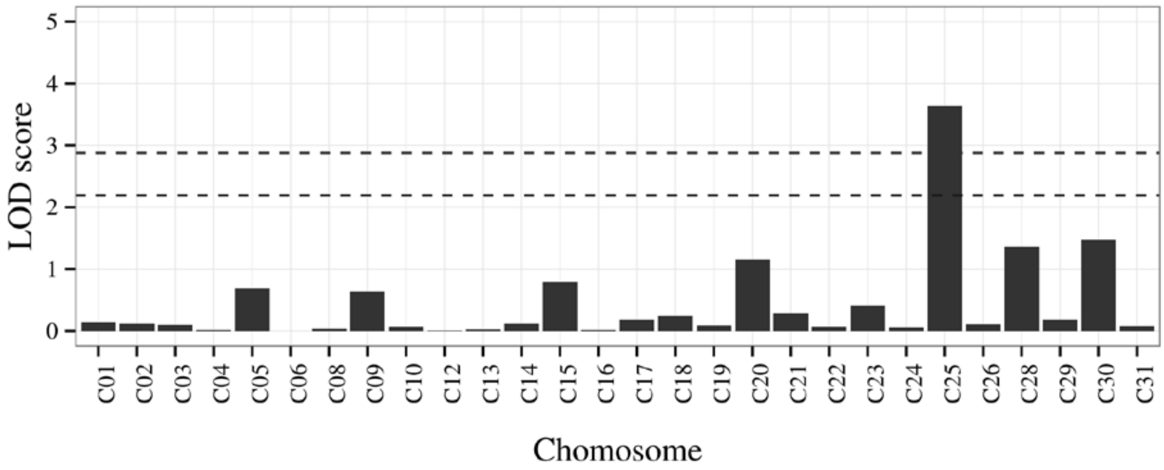

Additional file 11

LOD scores for all linkage groups in the combined analysis of the two timing backcross families, empirically determined by 10,000 permutations. 0.05 and 0.10 significance thresholds are represented by dashed lines.
