# Supplementary material for: Genetic basis of allochronic differentiation in the fall armyworm
Source: BMC Evol Biol. 2017 Mar 6;17:68. doi: 10.1186/s12862-017-0911-5 (PMC5339952; doi:10.1186/s12862-017-0911-5)
Supplement: Additional file 12: — Power analysis for backcross families. (PDF 317 kb) [file 12862_2017_911_MOESM12_ESM.pdf]

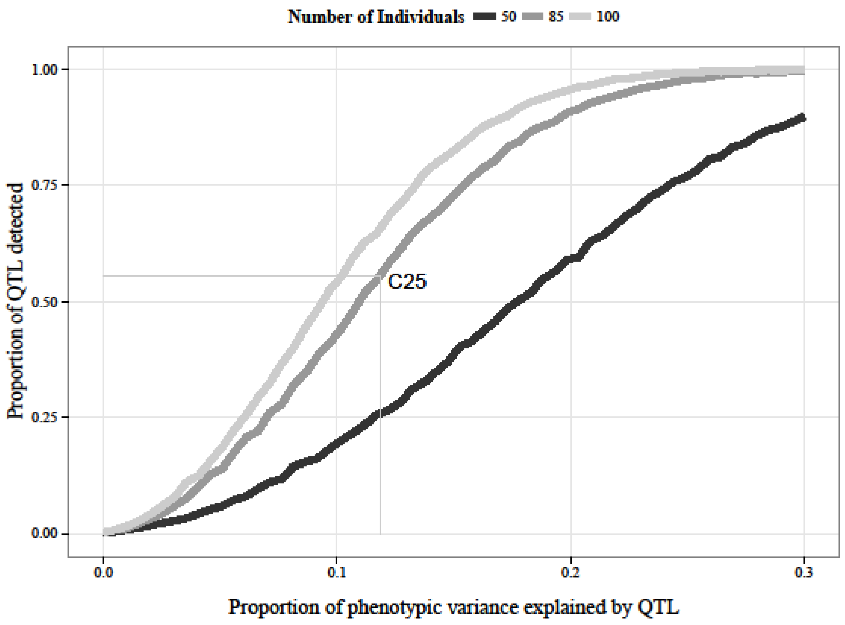

Additional file 12

Power analysis for backcross families with 50 (black line), 85 (corresponding to the QTL in this manuscript, dark grey line) and 100 (black line) progeny, respectively. The probability of detecting a QTL is plotted as a function of the fraction of phenotypic variance explained by the QTL.
