# Supplementary material for: Genetic basis of allochronic differentiation in the fall armyworm
Source: BMC Evol Biol. 2017 Mar 6;17:68. doi: 10.1186/s12862-017-0911-5 (PMC5339952; doi:10.1186/s12862-017-0911-5)
Supplement: Additional file 13: — Mating time in S. frugiperda hybrids. (PDF 29 kb) [file 12862_2017_911_MOESM13_ESM.pdf]

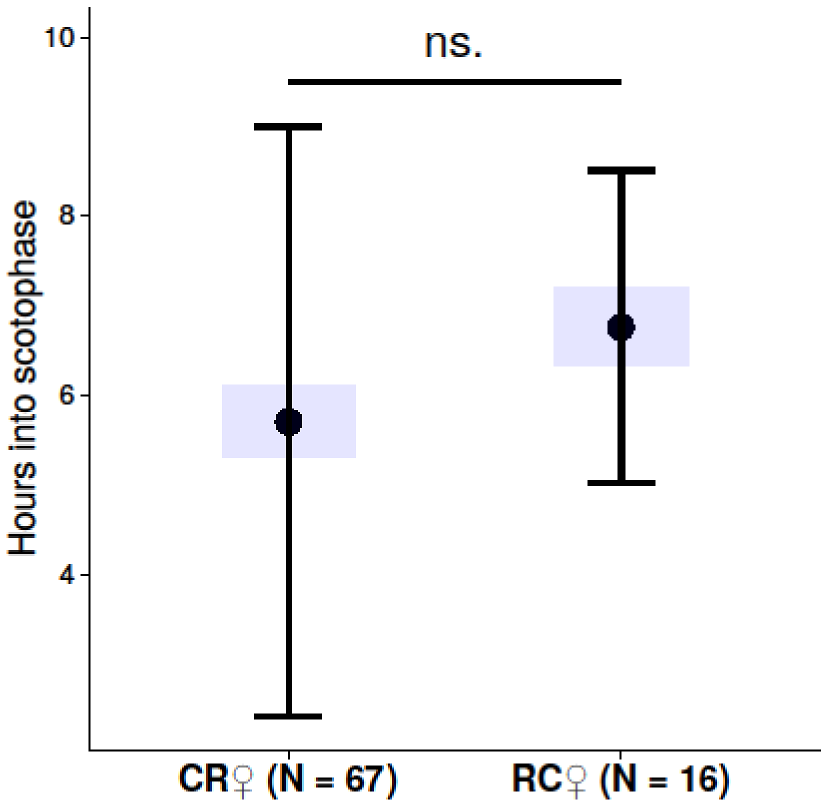

Additional file 13

Mating time in *S. frugiperda* hybrids.

Onset time of first mating in *S. frugiperda* hybrid females. The reciprocal crosses (CR= corn-strain mother, rice-strain father; RC= rice-strain mother, corn-strain father) do not show differences in mating time. This excludes the involvement of the sex chromosome in the timing differentiation.
