# Supplementary material for: Genetic basis of allochronic differentiation in the fall armyworm
Source: BMC Evol Biol. 2017 Mar 6;17:68. doi: 10.1186/s12862-017-0911-5 (PMC5339952; doi:10.1186/s12862-017-0911-5)
Supplement: Additional file 14: — Position of SNPs between the corn-strain and rice-strain of S. frugiperda in the coding regions of the major clock genes. (PDF 76 kb) [file 12862_2017_911_MOESM14_ESM.pdf]

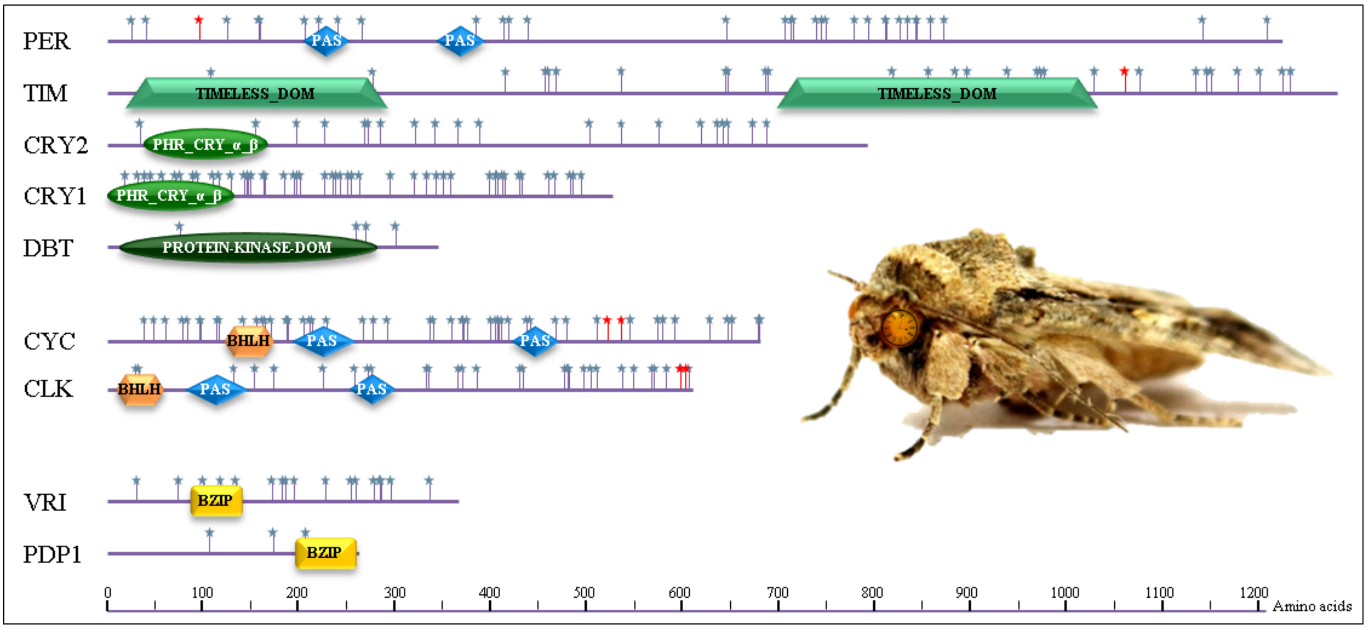

Additional file 14

Position of SNPs between the corn-strain and rice-strain of *S. frugiperda* in the coding regions of the major clock genes. Proteins are sorted by they position in the two different feedback loops (see Figure 1). Blue stars show synonymous SNPs, red stars show non-synonymous SNPs. Conserved protein domains are shown as colored shapes; same shape and name indicate the same domain function in different proteins. None of the non-synonymous SNPs are located in conserved domains of the proteins. PAS = PAS-domain (signal sensor), TIMELESS_DOM = TIMELESS specific conserved domain, PHR_CRY_α_β = Photolyase/cryptochrome alpha/beta domain, PROTEIN-KINASE-DOM = Protein kinase domain (with catalytic function), BHLH = basic helix-loop-helix (transcription factor family), BZIP = Basic leucine zipper (DNA binding).
